# A lightweight YOLOv11-based model for rice false smut detection under complex field conditions

**DOI:** 10.3389/fpls.2026.1808858

**Published:** 2026-05-28

**Authors:** Yingan Shao, Madhavi Devaraj

**Affiliations:** 1 School of Graduate Studies, Mapua University, Manila, Philippines; 2 School of Computer Science, Baicheng Normal University, Baicheng, China

**Keywords:** object detection, rice disease, YOLOv11, SCConv, GIoU

## Abstract

Rice false smut is an important fungal infection in the rice panicle stage, which occurs only in the panicle. Rice yield and quality will be seriously threatened after the occurrence of panicle disease. Early identification of disease is very important for precise prevention and control. However, in the actual field environments, complex light changes, the dense distribution of small disease spots, panicle overlapping shading, and other factors often result in the semantic attenuation of key discriminant information in the stage of visual feature extraction, which has brought great challenges to the early detection and prevention of the disease. To resolve the above problems, this study introduces a rice false smut detection model derived from an improved YOLOv11 framework, named Rice-Smut, to bolster the resilience and stability of the network regarding the identification of rice false smut disease under complex field backgrounds. Firstly, in order to enhance the feature capture capabilities for multi-scale and densely distributed lesions, the C3SC backbone feature extraction network combining the SCConv block is integrated. This architecture can significantly suppress the spatial and channel redundancy and augment the precise characterization of the texture features of the lesion. Then, the C2PSA-SE attention module is introduced to effectively filter the background interference and improve the precise positioning of dense small targets. Finally, to address the irregular structure of rice false smut lesions, the GIoU loss function serves as a substitute for the conventional CIoU, which enhances the network’s proficiency in locating the irregular shape lesions. Experimental outcomes revealed that the Rice-Smut model yielded a precision of 79.3% and mAP@50 of 75.3%, which represented a 7.6 and 4.5 percentage point improvement over the baseline model YOLOv11. The model requires 2.41M parameters, with a model size of 4.9MB, which results in low computational complexity. The preliminary validation on mobile platforms shows that the method is viable for the potential to be applied to the real-time field detection and disease monitoring of rice false smut, and can provide support for disease control decision-making and field management.

## 1 Introduction

Rice is a large scale consumed staple food source globally, and the yearly production of rice covers more than 50% of the global grain output. It is an important cash crop in numerous nations and major regions. Its production growth and quality bear a direct impact on economic stability and global food security (Ahad et al., 2023). However, rice is vulnerable to various fungal infections in the process of growth and development, and rice false smut is a prevalent and characteristic fungal malady in the panicle stage. The occurrence of rice false smut is susceptible to the impact of climatic conditions and rice varieties during the infection period, which may result in harvest deficits reaching as high as 70% in severe cases (Baite et al., 2021). Therefore, it is of great practical importance to implement early prevention and precise control of rice false smut.

The pathogen of rice false smut is Ustilaginoidea virens (Cooke) Takahashi, which is categorized as an ascomycete fungus. In the microstructure view, chlamydospores and conidia are the main vegetative structures of the strain. Chlamydospores are oval or spherical in shape, with a diameter of 4-6 μm, and exhibit dark green and tumor-like processes on the surface. Conidia are generated by chlamydospores after germination. The conidial stalk is short and small with septa, and can be solitary or branched. Multiple single-celled, oval or ovoid conidia are produced at the stalk end. In macroscopic morphology, the pathogen can form sporocarp structures after infecting rice panicles, and 1–4 long oval and flat sclerotia can be formed in the yellow area. The color of sclerotia gradually changes from white to orange-yellow during the development process, and finally changes to dark green or black, with a length of about 2–20 mm. After maturity, they fall off and overwinter in the soil. In the next year, under suitable environmental conditions, the sclerotia germinate and produce a fleshy stroma with a long stalk, the top of which is cap shaped or spherical, where the ascospores are released and the infection cycle was completed. The disease mainly occurs at the heading and flowering stage and is hidden. The ideal thermal condition for mycelial growth is 28-30 °C. After infection, it causes the emergence of orange-yellow or dark-green sclerotia in the glume, and also causes the production of ustiloxin and other metabolites, which seriously endanger rice quality and food security (Khanal et al., 2023). Timely detection and identification can provide scientific decision-making for the best application time through early precise identification of disease spots at each stage, especially at the early yellow stage, so as to avoid missing the best control window and leading to yield loss.

Lately, the swift advancement of machine learning, especially deep learning, has markedly enhanced the identification, real-time capabilities and intelligence level of rice pathology diagnostics. The rice disease detection methods based on machine learning are mainly used for the early identification of rice leaf, stem, root and seed diseases. At present, most researchers pay attention to image enhancement, noise removal and other preprocessing operations, and use computational algorithms including SVM, random forest, logistic regression, k-nearest neighbor, and Naive Bayes to classify rice diseases, as well as focused on K-means clustering, uniform sample selection algorithms and other methods for the segmentation and recognition of rice disease regions, achieving high detection performance in rice leaf disease recognition, which provides a useful reference for the intelligent diagnosis of rice diseases (Jiang et al., 2020). However, the above methods rely heavily on manually designed feature engineering, and need to extract features such as texture, color and shape with the help of expert experience. Especially in complex field environments, it is often difficult to guarantee the generalization ability of the model (Mukherjee et al., 2025). Especially for rice false smut, its early symptoms are not prominent, and the disease spots show complex morphological characteristics inside and outside the rice panicle shadows. At the same time, it is easy to be disturbed by light changes and rice panicle shading, which makes the occurrence of the disease highly hidden. Therefore, the above methods find it difficult to achieve precise identification of rice false smut, which may miss the best control opportunity and lead to the risk of excessive use of pesticides, further causing economic losses and environmental pollution.

Convolutional neural networks (CNN) built upon deep learning have seen extensive application across various domains. The automatic detection of plant diseases categories using CNN has gradually become the core topic in the current research field (Liu and Wang, 2021). Early studies mainly focused on image-level rice disease classification tasks, focusing on identifying the disease category of a single leaf or sample image. At present, most researchers use typical convolutional neural networks (such as ResNet, VGG16, etc.) to achieve high classification performance in rice disease recognition (Yusuf et al., 2024). Studies based on a multi-task learning framework combined with the VGG16 model have been utilized for the identification of agricultural foliage pathologies, and good recognition results have been achieved on datasets involving rice and wheat, demonstrating that the method has strong generalizability regarding the recognition of a variety of crop diseases (Jiang et al., 2021). Similarly, a variety of typical convolutional neural network models have been used to detect paddy infections and infestations, among which the VGG19 model performed the best, which further verifies the efficacy of this method in the task of complex crop disease identification, and provides a reference basis for disease monitoring and management in smart farming (Dey et al., 2022). In general, the methods built upon CNN have been widely studied in the task of rice disease classification, and has achieved relatively mature progress. However, such methods are usually accompanied by high computational costs, resulting in slow inference speeds. Especially importantly, these methods primarily aimed at the classification task of rice leaf diseases, but under complex field conditions, it is difficult to precisely locate and detect the disease spots with complex structures, dense distribution and vulnerability to background interference in rice false smut. Especially in the field of target recognition, Lately, in the research of two-stage target detection of rice diseases, most researchers have adopted a series of two-stage algorithms founded upon Faster R-CNN, Cascade R-CNN and so on to improve and optimize. While these techniques have achieved impressive results in detection precision, they rely on complex feature extraction mechanisms, which lead to high computational overhead and low inference efficiency, which limits their practical application ability in complex field environments. In contrast, in the research of one-stage rice disease target detection, efficient end-to-end detection characteristics have become the main research focus in the detection task of rice diseases, which is represented by the SSD and YOLO series one-stage algorithms, and continuous optimization is carried out in the optimization design of model structure and the improvement of feature expression. Existing literature indicates that it has become an important research trend to optimize and develop lightweight detection models under complex environmental conditions. To illustrate, (Liu et al., 2025) improved the YOLOv8n model to enhance the instantaneous diagnostic efficiency of rice diseases within intricate farming backgrounds. Aiming at the problem of complex background and diversification of lesion scales, (Fan et al., 2025) proposed a multi-level feature integration approach, which augmented the predictive stability and transferability of the model in complex field conditions. In addition, certain investigations prioritize the structural optimization of customized models for specific diseases (Pan et al., 2023). An improved rice bacterial blight model was proposed and achieved high precision. A lightweight rice detection model was designed for agricultural deployment scenarios (Zhou et al., 2023). At the same time, the SSD-based rice leaf model proposed by (Pan et al., 2025) has attained a harmonious between detection speed and precision.

However, in order to meet the challenge of detection in complex specific fields, researchers have proposed a series of improvement schemes. Specifically, Rose-Mamba-YOLO (You et al., 2025) and Succulent-YOLO (Li et al., 2025) respectively designed lightweight detection models for rose and succulent plant scenes, combining computer vision and UAV remote sensing technology to support real-time monitoring of plant growth in complex environments; Rose-YOLO (Zhao et al., 2025a) improves the detection ability of the rose growth cycle by integrating UAV, artificial intelligence and remote sensing technology; Mussel-YOLO combined with super-resolution reconstruction technology to enhance the robustness of freshwater mussel detection in complicated aquatic environments (Zhao et al., 2025b); Additionally, FP-YOLO is used for the instantaneous monitoring of floating plastic waste under complex water surface conditions (Liu et al., 2026). These studies also provide a reference for the present study in terms of the optimization strategies for detection tasks and model design for rice false smut within its complex background, multi-scale transformations, small target detection and resource constrained deployment.

While substantial advancements have been achieved regarding the localization and detection of rice leaf diseases, the field still faces numerous challenges under complex and changeable environmental conditions, including multi-scale changes of disease spots, occlusion, light fluctuations and background noise interference. These factors lead to the uncertainties in feature extraction, and further lead to the occurrence of false detections and missing detections, which limit the stability and generalization ability of existing detection models. Currently, related research is primarily centered on the identification of rice leaf diseases, while the research on the diseases occurring in the rice panicle, such as rice false smut, remains relatively limited, and there is a serious lack of the support from large-scale high-quality datasets. In addition, compared with the leaf area, the structure of the rice panicle area has a more complex three-dimensional spatial structure, and its disease spots are small targets, densely distributed, and are easier to be occluded, making the disease spots visually inconspicuous, which further increases the difficulty of detection. Therefore, under the premise of complex field environmental conditions and limited data samples, the effective detection of rice false smut remains of great practical significance and agricultural application value.

In order to overcome these challenges, this study constructed an improved detection model for rice false smut by utilizing the YOLOv11 target detection framework and optimizing it across the three dimensions: feature extraction, attention mechanisms and boundary regression. The main work of this paper includes: 1) combining with SCConv, a lightweight feature extraction module C3SC is designed. By replacing the standard C3k2 module, the channel and spatial redundancy are effectively reduced, the feature expression is enhanced, and the computational overhead is minimized. 2) The C2PSA-SE module is introduced to augment the network’s overall efficacy in precisely identifying fine lesions under complex background interference. 3) To improve the localization of irregular lesions, the GIoU loss function replaces the traditional CIoU, effectively avoiding the gradient interference, so as to achieve more stable bounding box regression. Experiments show that the model demonstrates reliability and stability on the current dataset. However, the recall rate for early tiny lesions still needs to be improved. The model can be applied to real-time monitoring of rice false smut under field conditions, and precise application of pesticide can provide auxiliary support for disease control decision-making. However, its generalization ability under the conditions of cross-region, multi-species and complex geographical conditions still needs to be further verified. In addition, through the preliminary verification of mobile applications, the practical utility value of this method in field real-time monitoring and disease prevention and control decision support is further demonstrated.

## 2 Materials and methods

### 2.1 Datasets

The images of rice false smut samples used in this study originated from several regions in Zhejiang Province, China, and were mainly collected in Wangjiangjing Town (Xiuzhou District, Jiaxing) and Xieqiao Town (Haining, Jiaxing). Data collection was carried out from July to September 2023, and the main cultivar was “zheyou 21”. The dataset covers various infection stages of the disease, ranging from the flowering stage to the grain filling stage, including samples across disease severity levels spanning the incipient, primary, and intermediate phases through to the terminal phases, so as to bolster the representativeness and variety of the sample data. These images were taken in the natural field environments using mobile phones, covering diverse lighting conditions, including strong light, weak light and shadow, as well as diverse shooting angles, leaf occlusion, panicle overlapping and other complex backgrounds. The original image resolution was 2448 × 3264 pixels, with a total of 1,963 disease samples. Since a single image usually contains multiple lesions, the total count of annotated targets is approximately 8,000. In the data preprocessing stage, the image orientation was automatically adjusted, and all samples were scaled to 640 × 640 pixels to comply with the input specifications of the neural network. Each lesion with a clear appearance boundary and independent visual features was annotated as an independent target instance, which eliminates the interference caused by occlusion and blurred boundaries. The Roboflow labeling tool was used to label the lesions professionally, and researchers with an agronomic background conducted the quality control, and the training, validation and test subsets were randomly according to a 7:2:1 distribution.

To clearly display the distribution patterns of the dataset, this paper shows the visualization results of the label statistics obtained during the training process. As shown in the instance count histogram (located in the top-left quadrant), although the dataset contains a constrained image volume, the total number of instances for the rice false smut category is close to 8,000, which provides sufficient data support for robust model training. The spatial distribution scatter diagram (lower-left corner) showed that the lesions had significant spatial variability in image coordinates. The bounding box size map (upper-right corner) shows that most lesions have significant differences in size and shape, showing multi-scale variation, mainly characterized by small objects. At the same time, the normalized width-height distribution (lower-right corner) shows that most target sizes are concentrated between 0 and 0.2 relative to the image scale. This covers a large number of small targets, as well as many medium-to large-scale targets.

**Figure 1:**
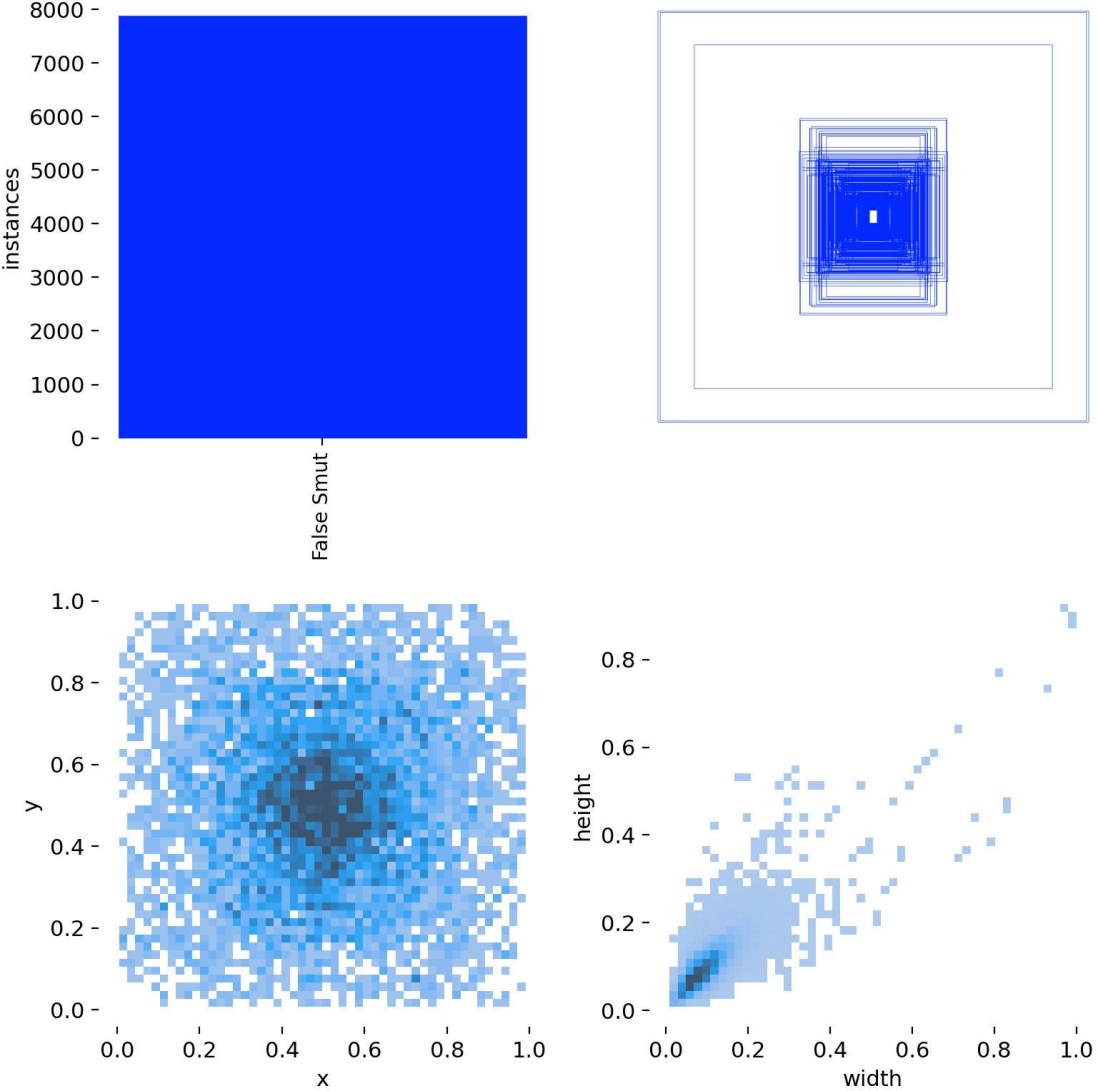
The label visualization results.

In accordance with the above complete analysis, the dataset exhibits diversity features in terms of density, multi-scale changes, and spatial distribution of target instances. This sample distribution can alleviate model overfitting of the model and can offer robust data support to augment the precision and reliability of the proposed approach. The visualization of dataset statistics is presented in **Figure 1**.

It should be noted that the samples in this dataset are mainly from a single geographical region. Although it covers a variety of environmental conditions, there are still some limitations in the generalization ability for cross-regional applications. Future work will further collect disease sample data from different regions to bolster the generalization capacity of the model. Additionally, because the sample size for the incipient phase is limited and difficult to capture, the number of samples in the early stage is insufficient, whereas the proportion of disease spot samples in the middle and late stages is relatively high, which may lead to a certain deviation in the recognition ability of the model for early-stage disease. Nevertheless, the disease characteristics of the four stages of rice false smut and the complex field environmental conditions covered by the dataset can more truly reflect actual field scenarios, and provide important data assistance for the automated surveillance and control of rice false smut.

### 2.2 Rice-smut module

This section systematically describes the overall architecture of the Rice-Smut detection model and its unified detection process, focusing on the analysis of the collaboration mechanism of the enhancement modules in natural scenes and their contributions to the enhancement of detection performance. From the perspective of practical agricultural applications, the operational mechanism of each module in the complex field environments and its supporting role in rice false smut detection task were analyzed. At present, the most advanced target recognition architectures mainly include the YOLO series built on CNNs and the RT-DETR series based on Transformers. YOLOv11 was released in September 2024 (He et al., 2024), with its model structure was optimized and improved, so that it made significant improvements in detection performance, cross-environment deployment, and a variety of visual tasks. In this paper, this study adopts YOLOv11 as the benchmark detection framework, which is structured to include three fundamental components: a feature extraction backbone, a multi-level feature fusion neck, and a specialized detection head.

Initially, the original sample image is convolved by the backbone network, and the important visual features of the lesion area are captured by feature extraction. In the actual complex farmland environments, due to light changes, panicle occlusion and background interference and other factors, the lesion area features show weak contrast and irregular visual distribution, which increases the difficulty of model detection. Aiming at these real challenges, such as multi-scale changes of lesions and complex background noise, this paper proposes the C3SC module, which integrates SCConv into the backbone structure. By using the channel reconstruction and spatial reconstruction mechanisms, the module effectively improves the feature capture capacity of the lesion region, optimizes the extraction efficiency, and improves the representational and capture abilities for multi-scale lesion features, which is more in line with the actual needs of agricultural monitoring. Subsequently, during the feature fusion block, for the purpose of improving the capability of the framework to detect densely distributed small-scale disease spots under natural field conditions, this study incorporated the SE attention block into the C2PSA module to construct a hybrid C2PSA-SE module. Adjusting channel weights help suppress the background interference caused by non-target features such as leaves and stems, and extract the key features of lesions, which is helpful to enhance the detection stability of the network in intricate field backgrounds. Finally, the detection head module classifies and locates the target of rice false smut. With the goal of boosting the localization precision of irregular shapes and small-scale regions, the GIoU loss function substitutes for the CIoU. This optimization scheme avoids the gradient vanishing phenomenon in the non-overlapping states, and improves the stability and precision of the bounding box regression for the densely distributed and irregular disease spots in rice panicles.

**Figure 2:**
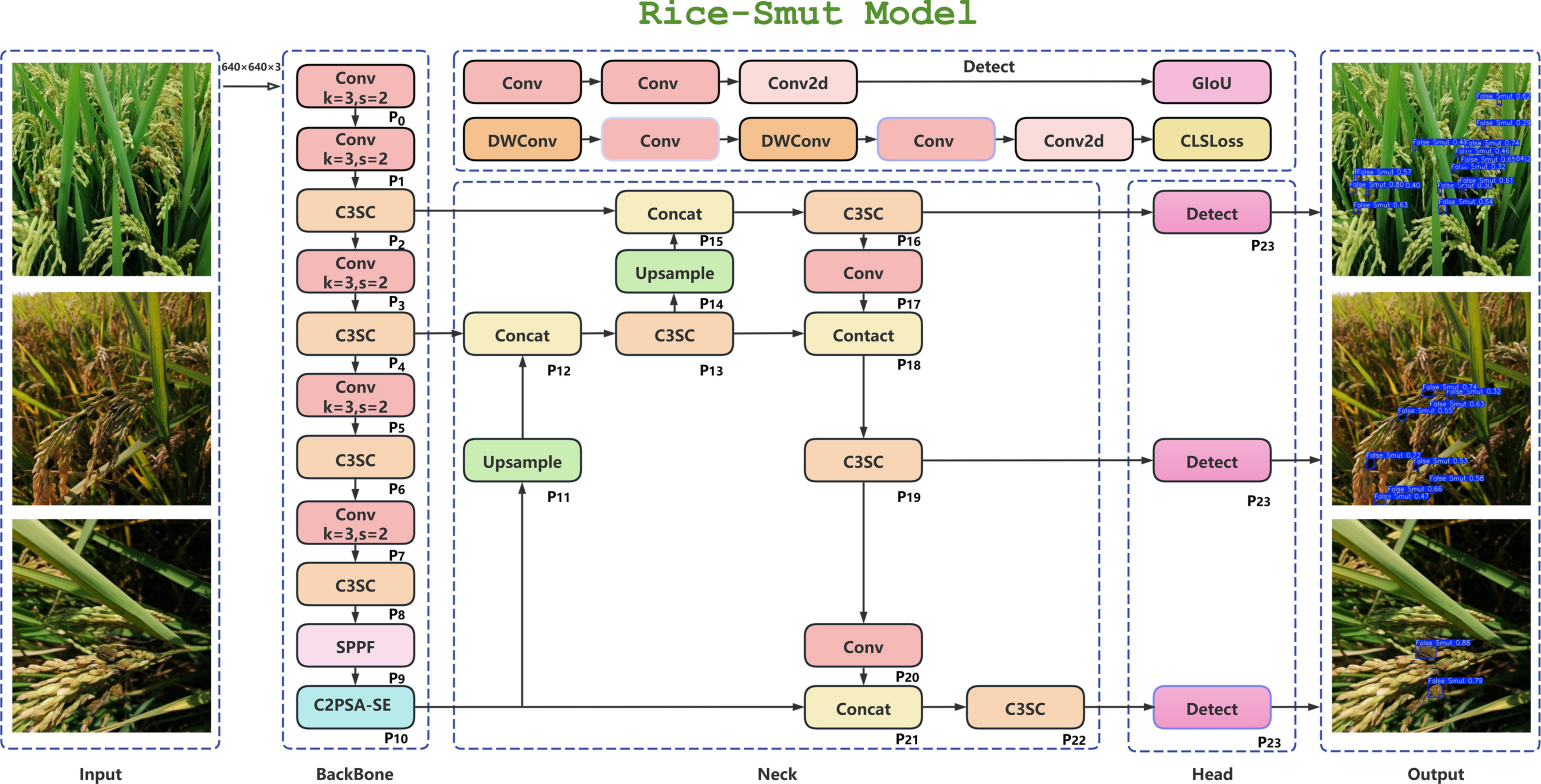
Illustration of the comprehensive pipeline of the Rice-Smut detection architecture.

The present study optimizes the architecture of the backbone hierarchy, integrates the attention block to enhance the fusion unit, and cooperates with the refined loss function to yield the collaborative improvement of the comprehensive performance of the network. The proposed architecture manifests substantial resilience for the identification of lesions in rice panicles under intricate field environments. The results of the model test can provide a direct basis for the assessment of the occurrence degree of rice false smut, so as to provide technical support for the precise application and decision-making of pesticides. This study provides an effective method of assistance for early-stage monitoring and precise positioning of rice false smut while taking into account the detection identification and computational efficiency. Concurrently, the model has the potential to be transplanted to mobile applications, and provides a feasible application scheme for real-time disease identification and monitoring in the field. The whole process is shown in **Figure 2**.

### 2.3 C3SC module

**Figure 3:**
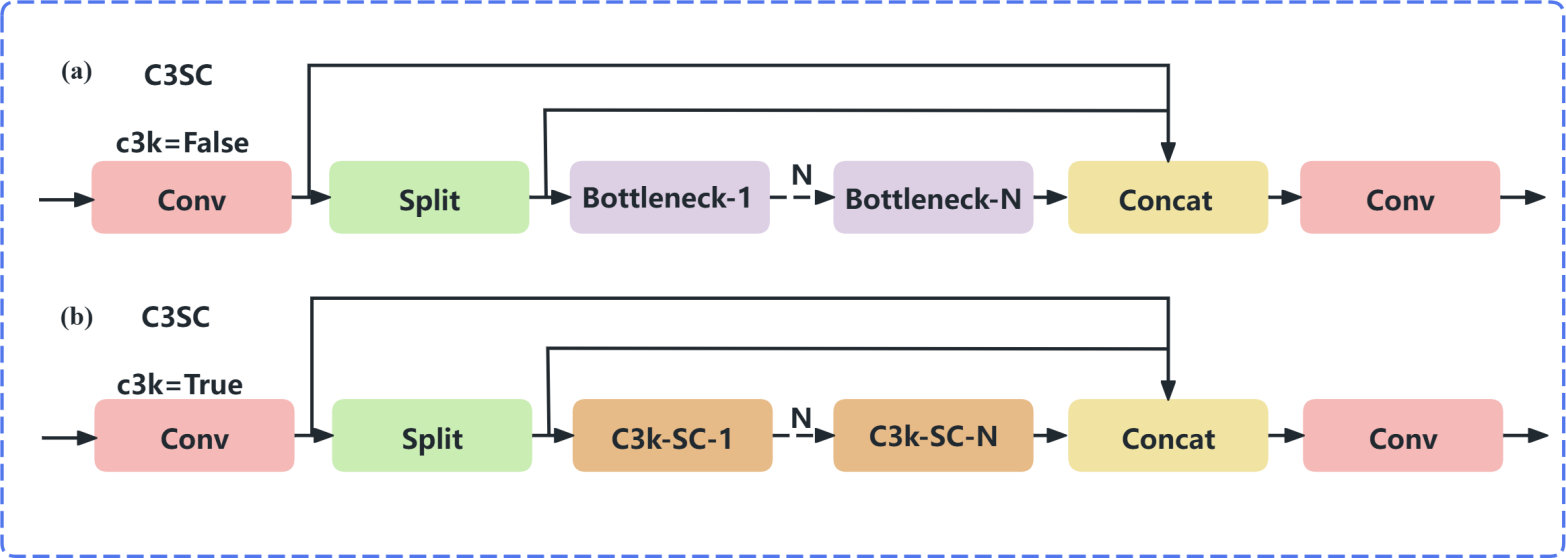
Architectural layouts of C3SC and its variants. **(A)** C3SC modules with c3k assigned as false; **(B)** C3SC modules with c3k assigned as true.

YOLOv11 redesigns the conventional C3 block by introducing the C3k2 block, which inherits the basic structure of the C2f unit. The structural composition of the C3k2 block is dynamically switched by a Boolean parameter (True or False); that is, the structural composition of the C3k2 module is selected between the C3k and C2f bottleneck structures. In the field environments, the disease spots of rice false smut usually show multi-scale changes, blurred boundaries and high similarity with the background, which imposes more stringent demands on the feature extraction capability for these lesions. The detailed architecture design of these modules is shown in **Figures 3A, B**.

**Figure 4:**
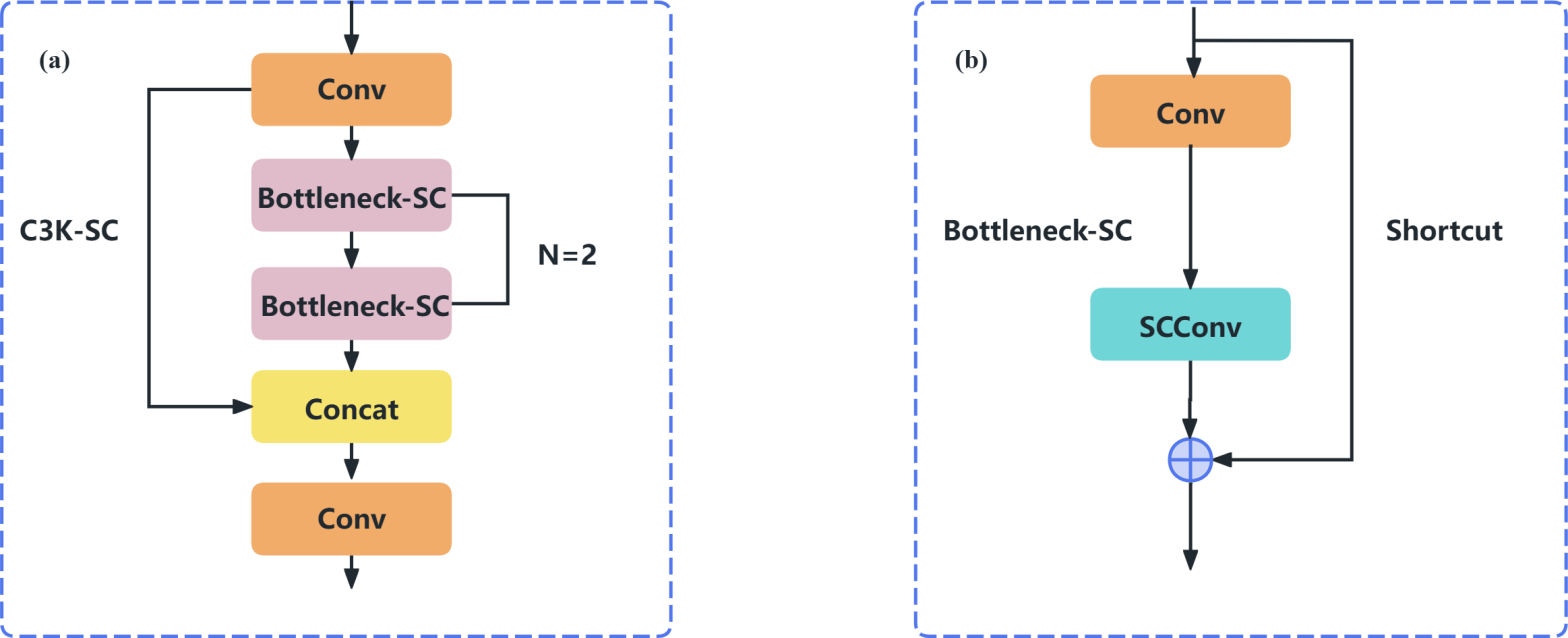
Architectural layouts of C3SC and its variants. **(A)** Architecture of the C3K-SC component; **(B)** Architecture of the Bottleneck-SC component.

The module uses adjustable convolution kernels, channel separation, and feature stitching technologies. This design can not only improve its ability to capture discriminant features, but also take into account the computational cost. When C3k=True, the proposed model applies the C3SC (C3k-SC) module by replacing the standard bottleneck unit in the C2f framework. Specifically, as shown in (**Figures 4A, B**), the SCConv operator is used to replace the second convolution layer of the bottleneck structure. This improvement helps to maintain the computational efficiency and bolster the capability of the network to perceive and capture features within the small lesion areas of rice panicles.

**Figure 5:**
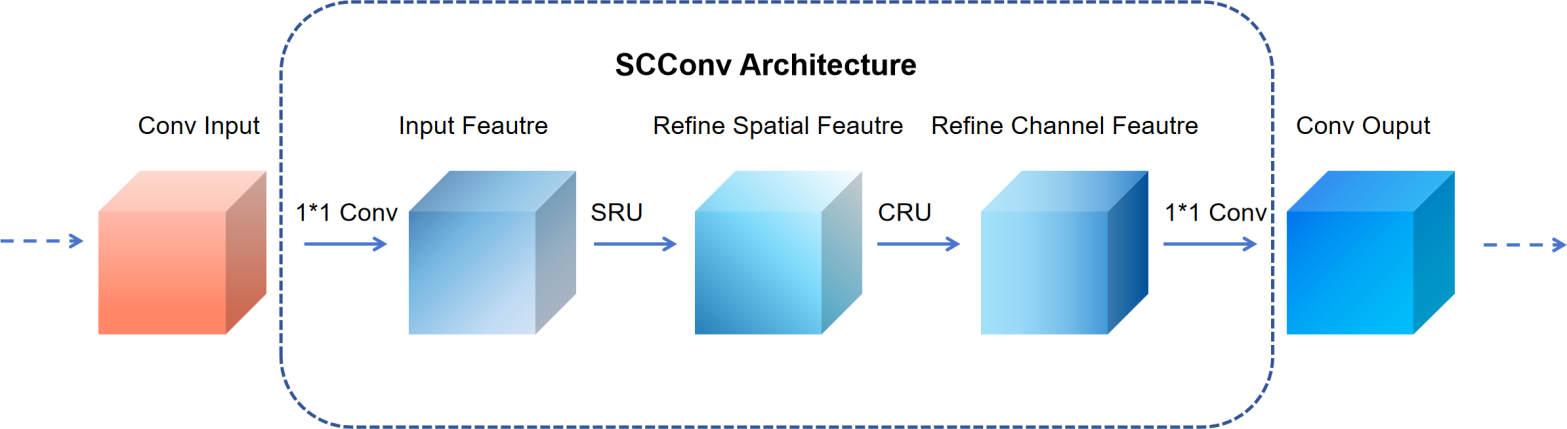
SCConv convolution block structure.

However, in practical applications, the sample data for rice false smut are usually collected in complex natural environments, which are characterized by dramatic changes in light, leaf occlusion, and diversity of shooting angles. Due to these factors, the network struggles to capture pathological details, which diminishes the precision of the network’s identification. To resolve these problems, this paper uses SCConv convolution operators instead of the standard bottleneck layer convolutions. SCConv consists of two primary modules, namely SRU and CRU (Li et al., 2023). This design can better mitigate the interference of complex field backgrounds, and help to enhance the predictive robustness of the framework in the field environment. The detailed framework is depicted in **Figure 5**.

**Figure 6:**
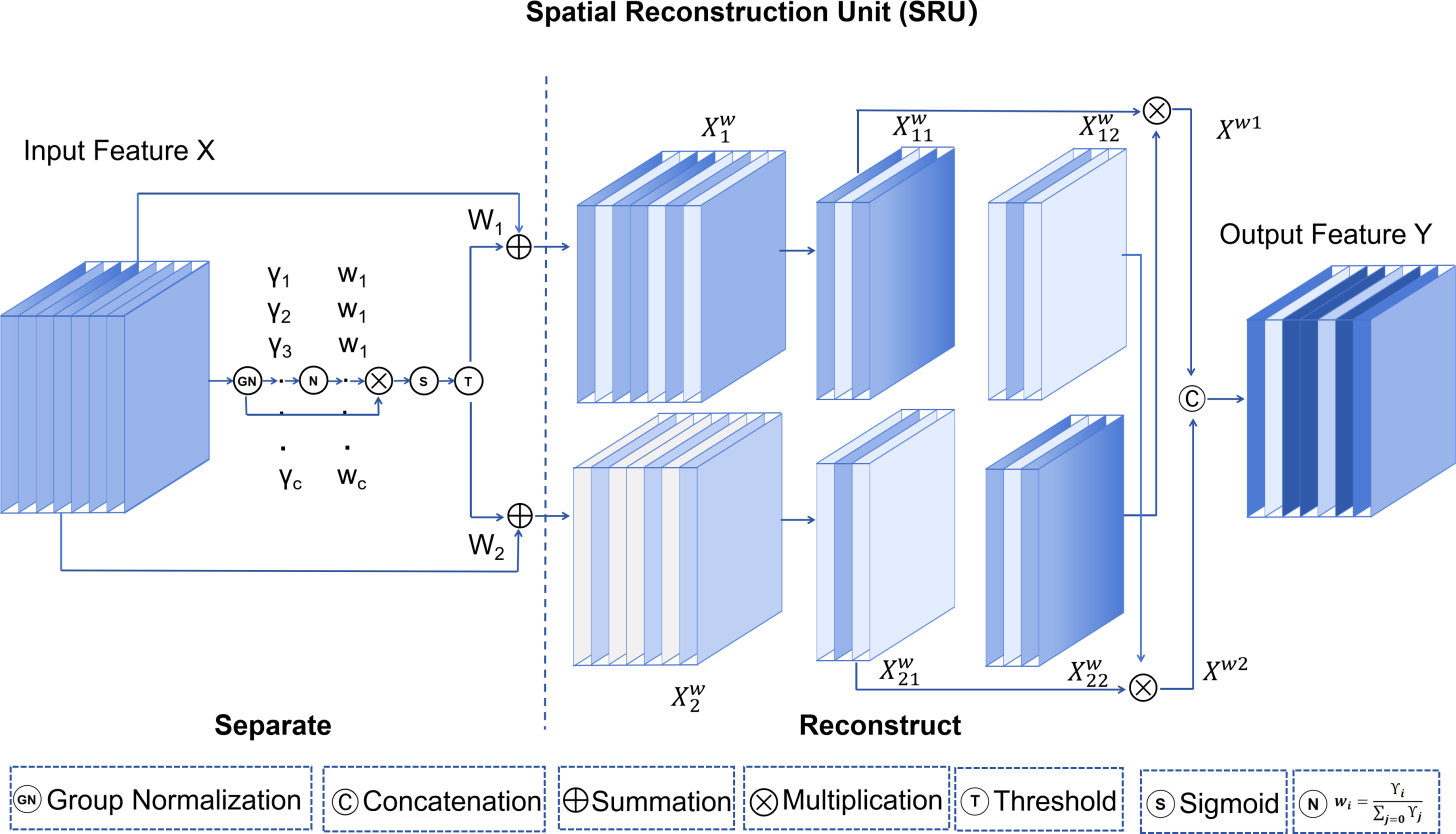
SRU module architecture.

Among them, the CRU first uses a 1 × 1 kernels to compress the input dimensionality from C to C/r (where r=2), then performs feature reconstruction, and finally restores the initial channel depth through another 1 × 1 kernel. This operation makes the convolution operation less computationally costly and proves effective in extracting lesion features. In order to decrease the redundancy of spatial features, the SRU is introduced, which includes two steps: separation and reconstruction. The filtering process can be used to separate regions with varying features successfully. This process helps to reduce the interference of complex field background noise, allowing the framework to focus more on the symptomatic indicators related to the disease. The fundamental structural design of this unit is illustrated in **Figure 6**.

To begin with, the input tensor $X\in {\mathbb{R}}^{N\times C\times H\times W}$ has four-dimensional tensors. In this case, N is the batch size, C indicates the number of channels, and H and W are the spatial dimensions. The specific processing procedure, including normalization, is given in **Equation 1**.

(1) $$\begin{array}{c} X_{out}=GN(X)=\gamma \frac{X-\mu }{\sqrt{\sigma^{2}+\epsilon }}+\beta \end{array}$$

In this case, the µ is the mean of the X, and σ is the standard deviation of the X, ϵ is a small constant added to guarantee numerical stability, while γ and β are the learnable parameters that are learned during the transformation process. The second step is important to calculate the scaling weights based on **Equation 2**.

(2) $$\begin{array}{c} W_{\gamma }=w_{i}=\frac{\gamma_{i}}{\sum_{j=1}^{C}\gamma_{j}},i,j=1,2,\ldots ,C \end{array}$$

Subsequently, the scaling factor W γ is mapped using a Sigmoid layer to derive the gating weights. Once the preset threshold is used, the gating weights are partitioned into informative weights $W_{1}$ and irrelevant weights $W_{2}$. This gating mechanism enables the architecture to filter the interference of non-target leaves and chaotic background noise, and highlight the characteristic information of the lesion area. The specific processing flow is displayed in **Equation 3**.

(3) $$\begin{array}{c} W=Gate(Sigmoid(W_{\gamma }(GN(X)))) \end{array}$$

Lastly, the element-wise product of $W_{1}$ and $W_{2}$, and the input feature X provides a rich and redundant representation of the features. The procedure is detailed in **Equations 4**–**8**.

(4) $$\begin{array}{c} X_{1}^{w}=W_{1}⊗X \end{array}$$

(5) $$\begin{array}{c} X_{2}^{w}=W_{2}⊗X \end{array}$$

(6) $$\begin{array}{c} X_{11}^{w}⊕X_{22}^{w}=X^{w1} \end{array}$$

(7) $$\begin{array}{c} X_{21}^{w}⊕X_{12}^{w}=X^{w2} \end{array}$$

(8) $$\begin{array}{c} X^{w1}\cup X^{w2}=X^{w} \end{array}$$

**Figure 7:**
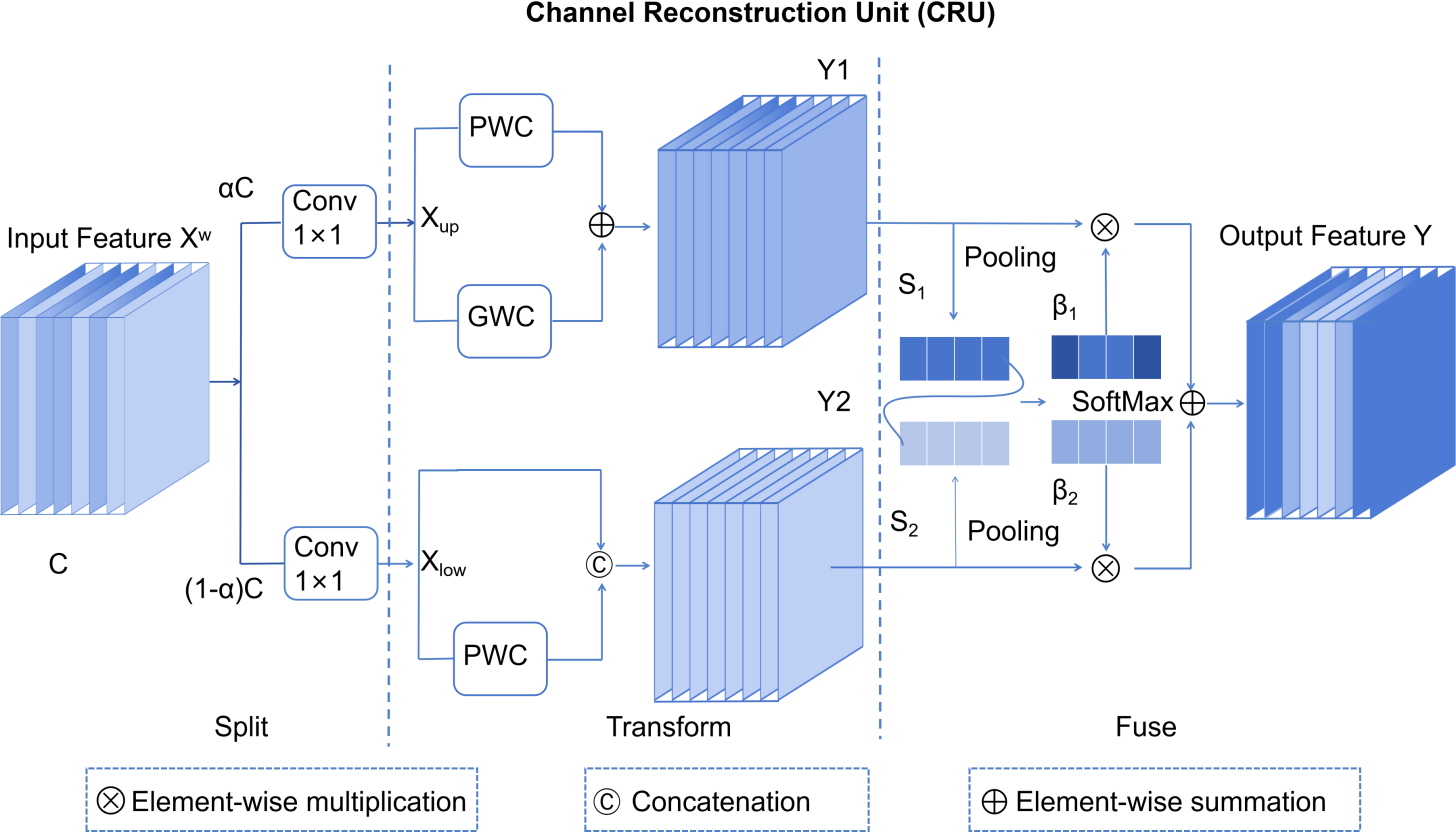
CRU module architecture.

To reduce channel redundancy, a Channel Redundancy Unit (CRU) is utilized. This unit consists of split, transformation, and fusion. In the transformation stage, grouped convolution (GWC) and pointwise convolution (PWC) were employed. This method minimizes the trainable parameters and computational resources of the network, as well as enhances the processing efficiency of the model. Through this lightweight design, the architecture is more feasible for real-time monitoring in the field environments, which provides technical feasibility for deployment. The architecture design of the CRU is detailed in **Figure 7**.

The main data processing flow of the CRU unit is as illustrated below: Firstly, divide the input feature space into two channels groups, αC, and (1-α) C. After a 1×1 convolution operation, two spatially enhanced features, X_up_ and X_low_ are obtained. Secondly, X_up_ and X_low_ values are obtained through the addition operations of GWC and PWC, respectively, the main processing procedure is shown in **Equations 9**, **10**.

(9) $$\begin{array}{c} Y_{1}=M^{G}X_{up}+M^{P_{1}}X_{up} \end{array}$$

(10) $$\begin{array}{c} Y_{2}=M^{P_{2}}X_{low}\cup X_{low} \end{array}$$

Finally, global channel description operators S_1_ and S_2_ are computed using global pooling on Y_1_ and Y_2_, Subsequently, the corresponding characteristic weights, β_1_ and β_2_, are generated, and the main processing procedure is shown in **Equations 11**–**13**.

(11) $$\begin{array}{c} \beta_{1}=\frac{e^{s_{1}}}{e^{s_{1}}+e^{s_{2}}} \end{array}$$

(12) $$\begin{array}{c} \beta_{2}=\frac{e^{s_{2}}}{e^{s_{1}}+e^{s_{2}}} \end{array}$$

(13) $$\begin{array}{c} \beta_{1}+\beta_{2}=1 \end{array}$$

After computing a weighted summation with Y_1_ and Y_2_, the weighted fusion mechanism enhances the recalibration of various feature channels, increases the weight of pathological features, and thus boosts the overall detection performance of the network. The main processing procedure is illustrated in **Equation 14**.

(14) $$\begin{array}{c} Y=\beta_{1}Y_{1}+\beta_{2}Y_{2} \end{array}$$

### 2.4 C2PSA-SE module

**Figure 8:**
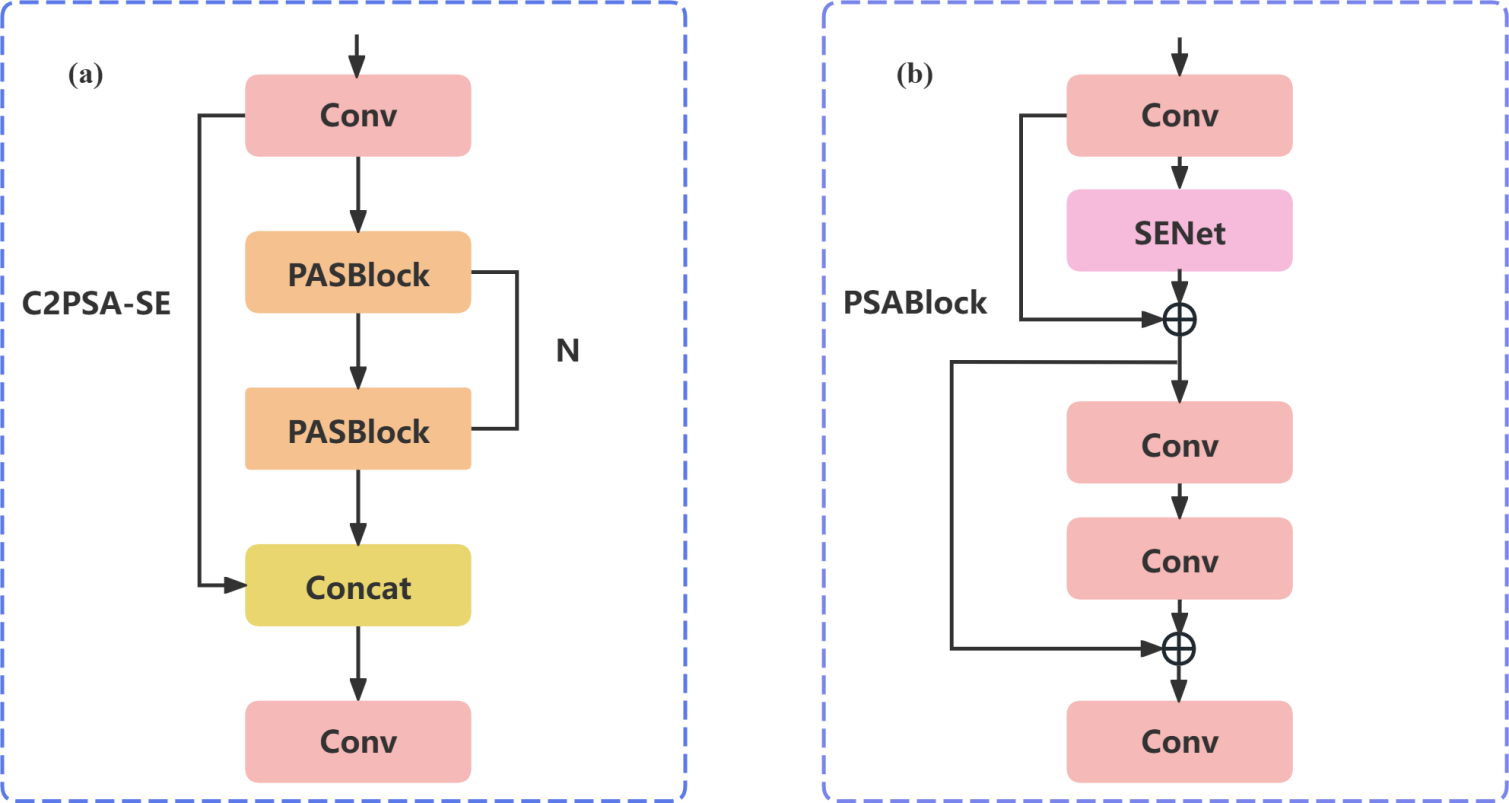
C2PSA-SE unit. **(A)** Architecture of the PSABlock component; **(B)** Architecture of the C2PSA-SE component.

The latest version of YOLOv11 introduces a brand new C2PSA component, which is located after the SPPF block. In order to further enhance feature learning in the backbone module, an MHSA block was integrated to form the C2PSA module. This is an important structural change in YOLOv11. The C2PSA module allows the model to simultaneously capture diverse feature information, better extracting features of pathological lesions. In the field environment, the disease spots often appear as small targets and are densely distributed, making them susceptible to interference from a variety of complex backgrounds, consequently, the model needs to have a better capacity for deep spatial representation and feature selection. The underlying design is shown in (**Figures 8A, B**).

In order to further improve the network’s capacity to recognize informative representations in disease recognition, the SENet attention mechanism (Hu et al., 2018) was introduced into the C2PSA module to construct the dependency relationship between channels in the feature representation of rice false smut disease. This can more effectively extract disease feature information, reduce noise interference, and strengthen the capability of the representation network.

**Figure 9:**
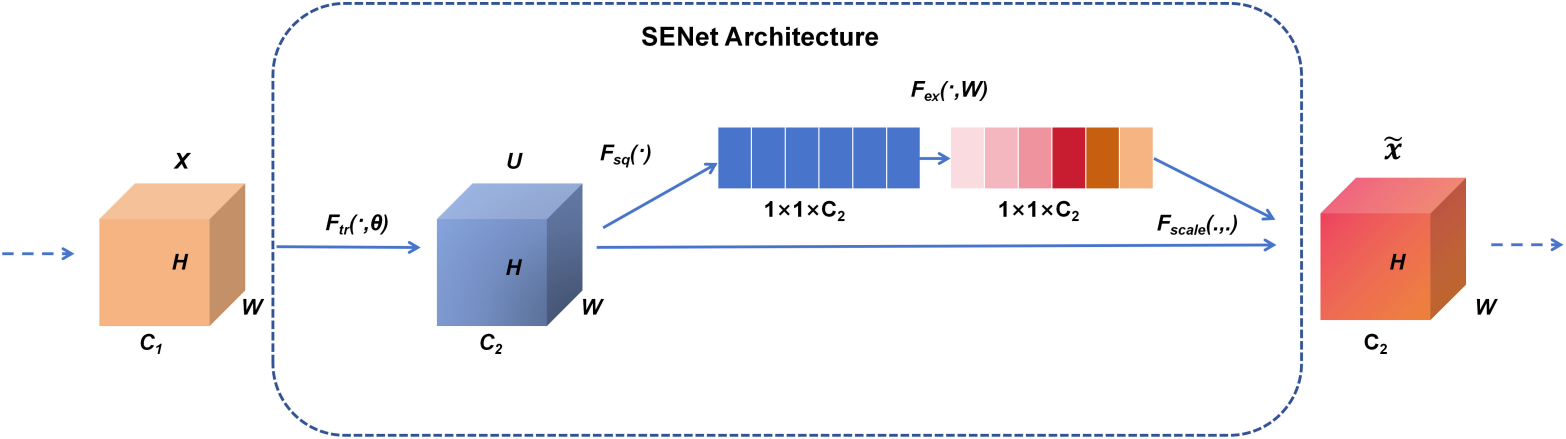
SENet module.

The SENet data processing flow consists of three key operations: Squeeze, Excitation, and Scale. Its fundamental model design is presented in **Figure 9**.

Step 1, The process initiates by passing the tensor X of the input C_1_ channel number through the Squeeze operation, and then performs the global average pooling operation to compress each channel into feature vectors. This process extracts the global spatial feature information, thereby enhancing the model’s perception of the overall lesion distribution characteristics. The corresponding mathematical expression is displayed in **Equation 15**.

(15) $$z_{c}=F_{sq}\left(u_{c}\right)=\frac{1}{H\times W}\sum_{i=1}^{H}\sum_{j=1}^{W}u_{c}\left(i,j\right)$$

Step 2, The Excitation operation is performed to learn a pair of dense layers and a ReLU activation operation, then, the weight score of the individual channel is attained by a using Sigmoid normalization layer. By adjusting the adaptive weights of the channels, the model pays more attention to the characteristic channels related to the disease. Its calculation is shown in **Equation 16**.

(16) $$\begin{array}{c} s=F_{ex}(z,W)=\sigma (W_{2}\delta (W_{1}z)),W_{1}\in {\mathbb{R}}^{\frac{G}{r}\times C},W_{2}\in {\mathbb{R}}^{C\times \frac{G}{r}} \end{array}$$

Step 3, The Scaling operation is performed to apply the attention weights of the input tensor. After applying the values, the final tensor is obtained. This operation effectively strengthens the response of key features of the disease, so as to reduce missed detection and improve the reliability in the detection of dense small targets, this is calculated as illustrated in **Equation 17**.

(17) $$\begin{array}{c} x_{c}=F_{scale}(u_{c},s_{c})=s_{c}\cdot u_{c} \end{array}$$

### 2.5 GIoU loss function

To facilitate the recognition of rice false smut in natural environments, where densely overlapping regions of the disease characteristics are often present. This is the key to quick and precise identification of the false smut. When the traditional IoU loss function is applied to two target objects with no overlapping regions, the IoU score is equal to zero, which fails to capture the relative spatial offset, and prevents the gradient optimization, meanwhile it is also difficult to effectively quantify the degree of overlap between targets, Therefore, designing and selecting an appropriate cost function can help promote the model’s detection precision.

YOLOv11 uses CIoU as the model’s loss function, where α helps balance positives samples and υ calculates the dimensional scale of identification results and the annotated enclosing boxes, c represents the extent of the box’s smallest diagonal, ρ calculates the geometric distance, the distance between the two box’s centers by b, and b^gt^ is also considered, as shown in **Equation 18**.

(18) $$\begin{array}{c} L_{CIoU}=1-IoU+\frac{\rho^{2}(b,b^{gt})}{c^{2}}+\alpha v \end{array}$$

Although the aspect ratio geometric constraints of the balanced samples have been taken into consideration, the attention to overlapping and non-overlapping regions is still insufficient. In particular, the early lesion areas of rice false smut are small and unevenly distributed. This can lead to missed detections during field detection of the model, which will interfere with the early prevention and decision-making for the disease. This study employs the generalized IoU metric (GIoU) loss function (Rezatofighi et al., 2019) instead of the standard IoU to enable the model to enable focus on both overlapping and non-overlapping regions. The main algorithm method is defined by: the intersection-union ratio is obtained by calculating the intersecting region of the boxes, and then the minimum bounding box of the two boxes is calculated. Thus, the model can still precisely locate and regress small-scale and densely distributed lesions. Experiments show that when locating dense, small-scale lesions, using GIoU as the optimization criterion shows better localization precision than the loss function using the traditional intersection-union ratio. Therefore, this method is more suitable for the identification and detection of rice false smut under complex field conditions. The last step is to compute the GIoU value using the subsequent equation, where A signifies the true-positive area and B denotes the localized prediction area. The variable C is defined as the area of the smallest enclosing box *C* encompassing *A* and *B*, The IoU denotes the overlap-to-union measure of *A* and *B*, as shown in **Equation 19**.

(19) $$\begin{array}{c} GIoU=IoU-\frac{\left|C-(A\cup B)\right|}{\left|C\right|} \end{array}$$

## 3 Results and analysis

To thoroughly analyze the validity of the improved model, this article first sets unified standards for environmental configuration and validation index parameter settings. Then, comparative experiments and ablation experiments based on different conditions are conducted. Finally, the effectiveness of each improvement module is tested through stability analysis and visualization of heat maps. At the same time, case studies of detection failures are introduced, which are divided into twelve dimensions to assess the overall model robustness and efficiency.

### 3.1 Computational environment configuration

The detailed introduction of the model experimental environment in this section mainly covers the selection of hardware equipment, the construction of the software environment, and the setting of hyperparameters. In order to ensure stable training of the model, this article relies on the Ubuntu 22.04 operating system and implements it under the PyTorch 2.1.0 framework in the development environment. In the deployment of computing resources, we chose an NVIDIA RTX 4090 GPU (24G) for its powerful floating-point computing capabilities, combined with 16 vCPU logic cores provided by an Intel Xeon Gold 6430 processor, and up to 120 GB of memory. The entire system greatly shortened the convergence cycle of the model through CUDA 12.1. In the data input stage, all data samples are set to a 3 × 640 × 640 format. In order to promote effective convergence of the model, this paper sets the training duration to 150 epochs. To achieve stability in model training, this study utilized a starting learning rate of 0.01, and accompanied by a batch size of 16. In addition, automatic mixed precision (AMP) is used during training, and finally, SGD is used for gradient calculation.

### 3.2 Evaluation criteria

The experimental performance was assessed using the following metrics: Precision (P), Recall (R), and the Mean Average Precision of the whole class when the threshold of the intersection and merge ratio was 0.5 (mAP@50), GFLOPs, model size (MB), frame rate (FPS), and parameters (Params). This paper uses several evaluation metrics to validate the model’s capability, consisting of Precision, Recall, mAP@50, and mAP@50-90. The comprehensive performance of the model is assessed using the above indicators. The corresponding calculation formulas are provided in **Equations 20**–**22**.

(20) $$\begin{array}{c} P=\frac{TP}{TP+FP} \end{array}$$

(21) $$\begin{array}{c} R=\frac{TP}{TP+FN} \end{array}$$

(22) $$\begin{array}{c} mAP=\frac{1}{N}\sum_{i=1}^{N}AP_{i} \end{array}$$

In the above evaluation metrics, TP, TN, FP, and FN respectively denote the numbers of true positives, true negatives, false positives, and false negatives. N is used to denote the number of classes of objects, and $AP_{i}$ denotes the average precision of the i-th class.

### 3.3 Comparative experiment

To ensure that the performance of all models (including YOLOv5, YOLOv8, YOLOv9-t, YOLOv10n, YOLOv11, YOLOv12 (Tian et al., 2025), and the proposed Rice-Smut) can be fairly compared, we followed three key steps. Firstly, a unified set of hyperparameters was adopted to eliminate the differential effects caused by manual optimization; secondly, standardized hardware and software configuration; finally, all networks were initialized without using any pre-trained weights.

As illustrated in **Table 1**, contrasted with other baseline models, the proposed Rice-Smut model achieved an increase in mAP@50 of 4.8%, 2.8%, 3.8%, 5.5%, 4.5% and 4.4%, respectively. In terms of model efficiency, the number of parameters was reduced by 4.6 M, 0.6 M, 0.39 M, 0.28 M, 0.17 M, and 0.15 M, correspondingly. The model requires only 4.9 MB of memory space and 2.41 M parameters, making it the most compact model among all test versions. The experimental findings show that the Rice-Smut model can efficiently improve the feature extraction capability for disease spots while maintaining low computational overhead. Especially in the field conditions with dense distributed rice panicles and similar textures, this feature enhancement mechanism helps to distinguish between lesions and background noise, so as to enhance the discrimination capability of the model.

**Table 1:** Comparative experimental indicators of various models.

Model	P	R	mAP@50	Size	GFLOPs	FPS	Params
YOLOv5	70.4%	70.7%	70.5%	13.8	15.8	77.3	7.01
YOLOv8	74.5%	65.4%	72.5%	6.0	8.1	136.8	3.01
YOLOv9-T	72.9%	64.5%	71.5%	23.5	11.7	45	2.80
YOLOv10n	73.6%	66.2%	69.8%	5.5	8.2	99.6	2.69
YOLOv11	71.7%	68.7%	70.8%	5.2	6.3	143.6	2.58
YOLOv12	72.9%	63.9%	70.9%	5.3	6.3	104.1	2.56
Rice-Smut(**ours**)	**79.3%**	67.6%	**75.3%**	**4.9**	**6.2**	82.6	**2.41**

The bold values indicate the best-performing results for each evaluation metric.

**Figure 10:**
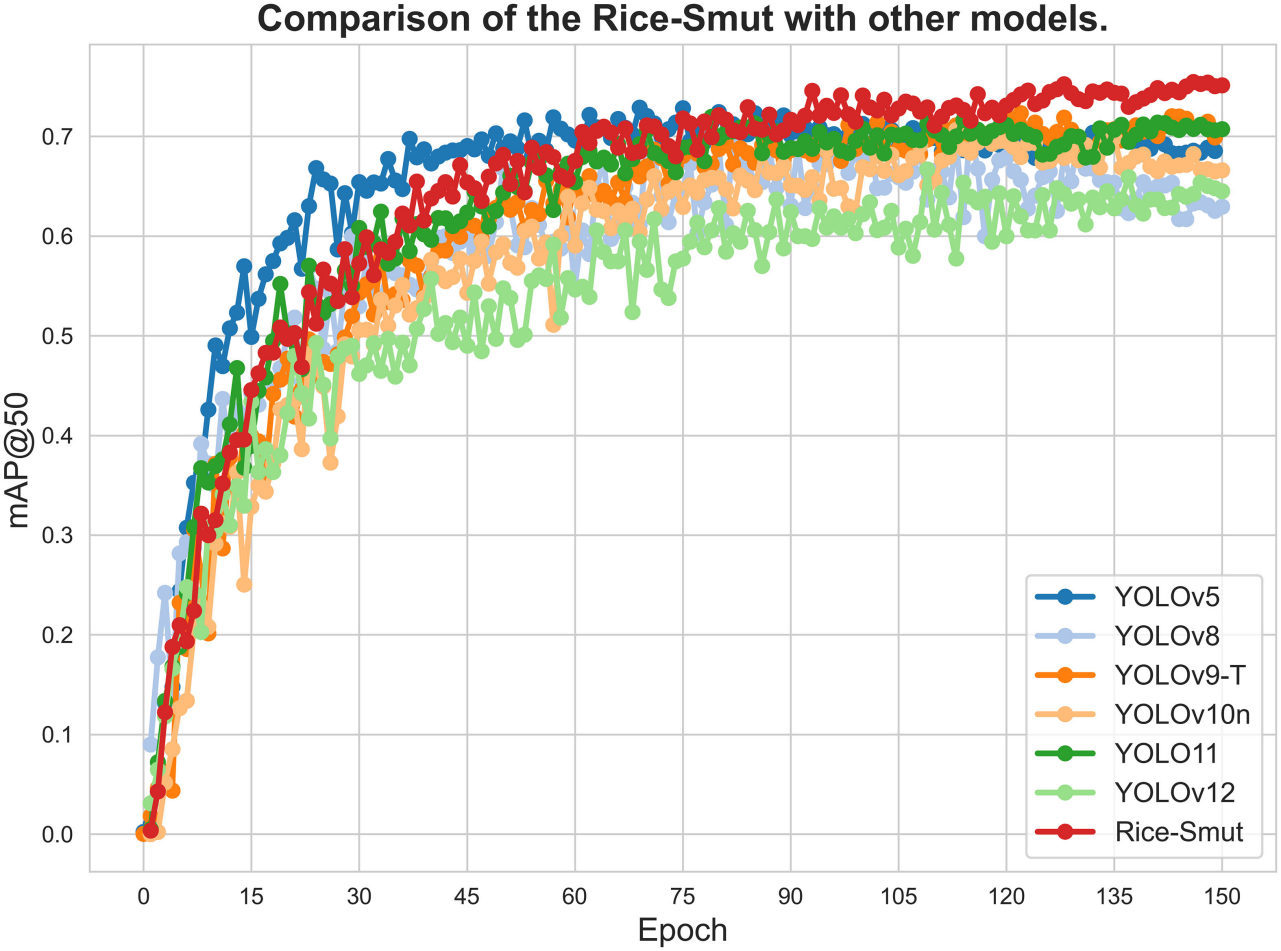
Comparison of mAP variation curves between Rice-Smut and other models.

Subsequently, this study made a comparison of the results of each model’s mAP@50. The indicators were evaluated and quantified, with the findings are illustrated in **Figure 10**. During the first 30 epochs of training, YOLOv5, YOLOv8, YOLOv11, and Rice-Smut all showed fast convergence speed, and the performance of YOLOv5 improved most rapidly. After training for more than 100 epochs, the Rice-Smut model’s mAP@50 keeps growing and is always better than other comparison models. This demonstrates that the model has significant performance advantages in target localization and classification tasks. From a practical application perspective, augmenting the network’s capacity to represent the features of dense, small lesions contributes to improved recognition stability in field environments; this ensures that, even during the later stages of training, the model remains sensitive to subtle pathological changes, thereby boosting the reliability of disease monitoring.

### 3.4 Experimental comparison of training metrics for baseline models

**Figure 11:**
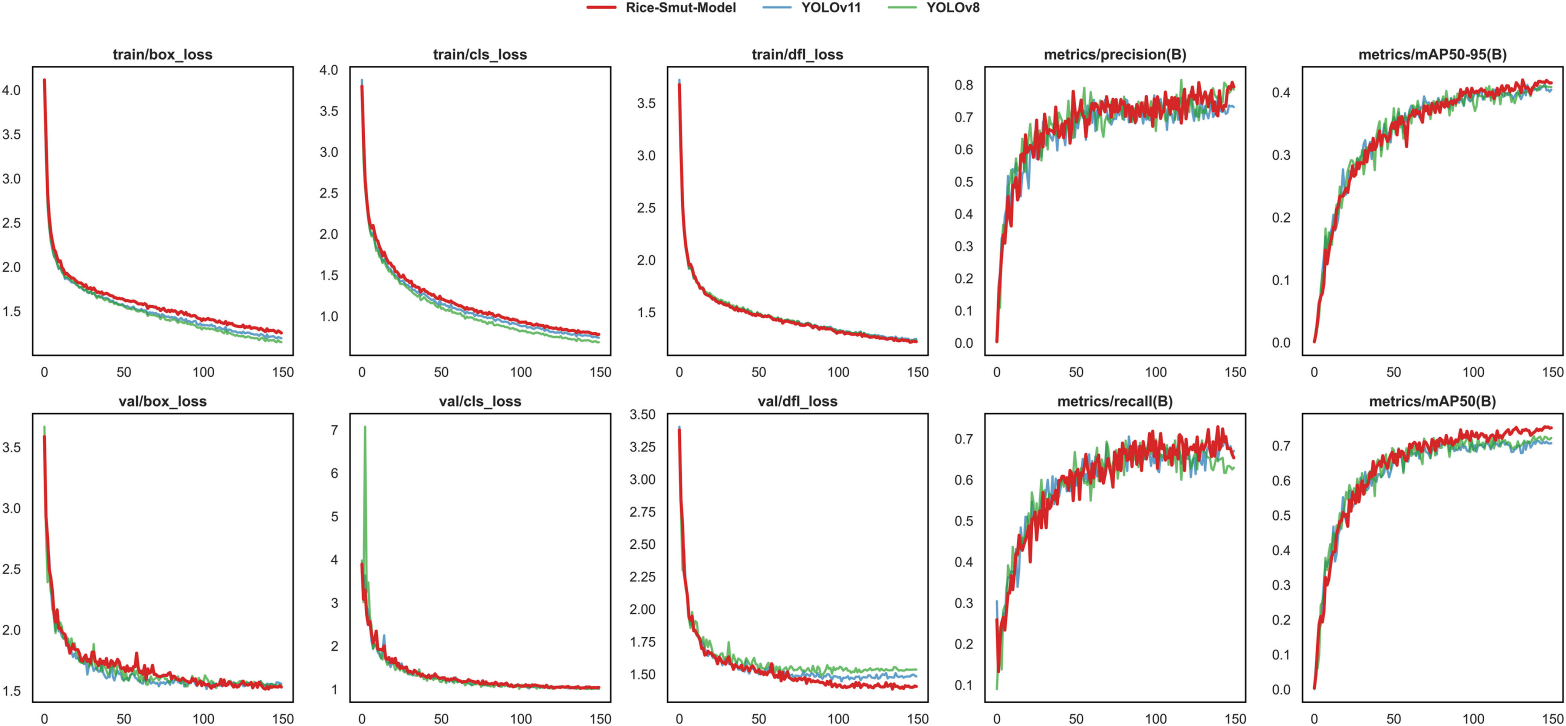
Comparison of training metrics curves between the Rice-Smut and other models.

In this paper, the important baseline models YOLOv8, YOLOv11 and the improved Rice-Smut model are used to comprehensively compare the training metrics. First, the Rice-Smut model achieves the highest mAP@50, with a value of 75.3%, which is significantly better than YOLOv8 (72.5%) and YOLOv11 (70.8%). The results show that the improved model has better feature extraction capabilities in complex field environments, and can effectively distinguish between the diseased and non-diseased areas. This is of great practical value for the identification of lesions with similar colors, textures, and dense distributions in rice false smut. By observing the trend of the convergence curves, it can be found that the network shows a more stable performance improvement in the late stages of training, which indicates that it has a stronger advantage in the feature extraction of disease spots. Secondly, regarding the precision metric, the detection precision of the Rice-Smut model is as high as 79.3%, which outperforms all comparison models. In addition, with respect to recall indicators, the YOLOv5 model performed best, significantly ahead of all comparison models, while the Rice-Smut model reached 67.6%, remaining at the upper-middle level. Although this recall rate is still at a moderate level, it shows that there are still some missed detections persist in the complex natural field environments, which may affect the early warning capability in the actual disease monitoring. The main reason is that the sample distribution of the dataset is unbalanced and the interference of various complex noises hinders the model to fully learn the symptomatic expressions of each stage under all conditions. Finally, in terms of model size, the Rice-Smut model is the smallest among all evaluated models, with lower memory consumption and minimal inference latency, which is convenient for implementation, as shown in **Figure 11**.

### 3.5 Ablation experiment

Three independent replicates were carried out under different random seed initialization conditions. The findings are presented as the statistical mean ± standard deviation, so as to ensure the stability and statistical robustness of the conclusions. This study uses YOLOv11 as the benchmark architecture, and uses Precision (P), Recall (R), mAP@50, model size, GFLOPs, frame rate, and total parameters as evaluation indicators to validate the effectiveness of the optimized modules. The study carried out ablation experiments on different block combinations. Firstly, the C3SC module constructed with the improved C3k2 replaced the standard C3k2 in the backbone and neck modules, which improved the model Precision by 2.3% and mAP@50 by 3.3 percentage points. The experimental findings show that the SCConv structure can operatively augment the capture ability for multi-scale lesion features, especially suitable for dense small targets within complex field environments. Secondly, aiming to bolster the recognition effect of rice false smut spots in complex natural environments, we introduced the C2PSA-SE model, increasing mAP@50 by 1.7 percentage points. This results that the introduction of attention module can effectively suppress the interference of complex background clutter such as leaves, panicles, and occlusion, and improve the feature representation capability of the model for the lesion areas. Then, after substituting the initial CIoU loss function with GIoU, the network’s Precision and mAP@50 were further improved by 6.5% and 1.3%, respectively. This improvement is helpful to enhance the localization capability of the network in the case of non-overlapping targets, so as to reduce the positioning error of multi-scale disease spots, especially for scenes with sparse distribution or irregular and dense boundaries of rice false smut. Finally, compared with the benchmark network, the improved Rice-Smut model has outperformed it with respect to Precision and mAP@50 (increasing by 7.6% and 4.5%, respectively). Simultaneously, the network size was decreased by 0.3 MB, and the parameters were minimized by 0.17 M. These experiments indicate that the model is able to effectively detect rice false smut. The findings are presented in **Table 2**.

**Table 2:** Component-wise ablation analysis based on the baseline configuration.

C3SC	C2PSA-SE	GIoU	P	R	mAP@50	Size	GFLOPs	FPS	Params
−	−	−	0.728 ± 0.022	0.672 ± 0.016	0.712 ± 0.010	5.2	6.3	143.6	2.58
✓	−	−	0.754 ± 0.048	0.669 ± 0.037	0.725 ± 0.015	5.0	6.2	81.6	2.45
−	✓	−	0.745 ± 0.023	0.677 ± 0.022	0.718 ± 0.005	5.1	6.3	132.0	2.53
−	−	✓	0.754 ± 0.029	0.652 ± 0.014	0.712 ± 0.010	5.2	6.3	128.3	2.58
✓	✓	−	0.752 ± 0.037	0.679 ± 0.010	0.737 ± 0.009	4.9	6.2	80.1	2.41
✓	✓	✓	0.759 ± 0.043	0.682 ± 0.005	0.740 ± 0.013	4.9	6.2	82.6	2.41

### 3.6 Ablation study of SCConv in the C3K2 block

In this study, seven mainstream convolutional layers are selected for horizontal comparison in view of the effectiveness of feature extraction for lesions. The first type is based on dynamic convolution, including ODConv and SAConv to improve the multi-level feature extraction of complex disease spots. The second type is based on the convolution of attention and receptive fields, such as RFAConv, RepVGG, and MAB. This category of convolution focuses on the attention mechanisms and parameter optimization to improve the perception of disease spots. The third type, lightweight convolution, such as SCConv and FasterNet, achieves a balance between cost and efficiency by reducing the operational costs and simplifying the convolution operations.

The C3k2 module has been extensively improved. All corresponding components in the backbone and neck networks have been replaced. Experimental data show that the improved C3k2 structure based on SCConv achieved the best mAP@50 metric, reaching 74.1%. This shows that the design of a lightweight convolution structure can help the model to sustain strong feature representations ability while reducing the computational complexity. It is suitable for hardware-limited agricultural applications environments under the premise of balancing the detection precision and efficiency. The above findings indicate that the improved network has stronger robustness and representational capacity in the feature extraction of rice false smut, as shown in **Table 3**.

**Table 3:** Ablation experiment of different convolutional alternatives in the C3K2 block (including SCConv).

Configuration	mAP@50	Size	GFLOPs	FPS	Params
C3k2	70.8%	5.2	6.3	143.6	2.58
C3k2+MAB	70.3%	5.3	6.6	89.3	2.60
C3k2+RFAConv	71.0%	5.4	6.6	75.5	2.63
C3k2+ODConv	72.3%	6.5	5.9	80.4	3.25
C3k2+FasterBlock	72.8%	5.4	5.1	134.8	2.54
C3k2+RepVGG	72.9%	5.3	6.4	118.6	2.61
C3k2+SAConv	73.8%	5.8	5.9	82.1	2.85
C3k2+SCConv(**C3SC**)	**74.1%**	**5.0**	6.2	81.6	**2.45**

The bold values indicate the best-performing results for each evaluation metric.

### 3.7 Comparative experiment of attention mechanism in C2PSA module

To augment the performance of the backbone architecture in detecting the characteristics of rice false smut, the C2PSA module introduces a strengthened scheme incorporating the attention mechanisms, which leads to improved detection performance in complex scenes. Recently, seven types of attention mechanisms were selected for evaluation, including SENet, Triplet, EMA, MSDA, CGA, CAA, CBAM, and SEAM.

**Table 4** shows that CAA is relatively balanced in all aspects, but its mAP@50 value is the lowest because it mainly focuses on long-range pixel dependencies. Triplet can significantly improve the detection performance by selecting three different dimensions, but the recall rate is still low. EMA, MSDA, and CGA focus on multi-scale features, and all attention mechanisms have similar mAP@50 values. At the same time, CBAM, which pays attention to the channel characteristics first and then makes spatial selection, is insufficient to capture the lesion characteristics of rice false smut.

**Table 4:** Performance comparison results of C2PSA variants with different attention mechanisms).

Configuration	P	R	mAP@50	Size	Params
C3SC+C2PSA+CAA	75.9%	64.8%	71.7%	4.7	2.31
C3SC+C2PSA+Triplet	77.7%	66.7%	72.1%	4.7	2.27
C3SC+C2PSA+EMA	73.8%	67.3%	72.9%	4.7	2.27
C3SC+C2PSA+MSDA	75.9%	69.0%	73.2%	5.0	2.47
C3SC+C2PSA+CGA	76.5%	67.7%	73.3%	4.8	2.30
C3SC+C2PSA+CBAM	72.8%	68.2%	72.3%	5.2	2.42
C3SC+C2PSA+SEAM	76.7%	66.8%	73.9%	4.9	2.42
C3SC+C2PSA+SENet(C2PSA-SE)	**77.3%**	66.8%	**74.5%**	4.9	2.41

The bold values indicate the best-performing results for each evaluation metric.

The SEAM attention mechanism focuses on improving the problem of target occlusion, and its mAP@50 value is 73.9%, which outperforms the multi-scale attention mechanisms. Finally, the research shows that the classic SENet channel attention mechanism performs best, reaching a mAP@50 value of 74.5%. The experimental findings indicate that the introduction of a channel attention module is beneficial for augmenting the characteristics of rice false smut, while the use of more complex attention structures does not make the model achieve better performance improvement. This shows that the attention mechanisms with simple structures and high efficiency have more greater practical application potential in the detection of rice false smut.

### 3.8 Comparative experiment based on the GIoU loss function

This paper selects seven loss functions for evaluation, including DIoU, CIoU, GIoU, SIoU, EIoU, Focal-EIoU, and UIoU. Among them, Focal-EIoU yielded the lowest mAP@50 value (54.4%), while the GIoU achieved a mAP@50 of 67.6%, which also reflects the overall recall rate performance. The experimental comparison outcomes are reported in **Table 5**.

**Table 5:** Comparative experiment of different boundary box regression loss functions based on C3SC+C2PSA-SE framework.

Configuration	P	R	mAP@50
C3SC+C2PSA-SE+FocalUIoU	57.2%	52.7%	54.4%
C3SC+C2PSA-SE+UIoU	72.1%	66.3%	69.0%
C3SC+C2PSA-SE+EIoU	79.4%	61.7%	70.9%
C3SC+C2PSA-SE+SIoU	78.4%	62.3%	71.1%
C3SC+C2PSA-SE+CIoU	71.7%	68.7%	70.8%
C3SC+C2PSA-SE+DIoU	72.9%	68.0%	72.9%
C3SC+C2PSA-SE+GIoU	**79.3%**	67.6%	**75.3%**

The bold values indicate the best-performing results for each evaluation metric.

This shows that GIoU can provide a more stable optimization gradient when dealing with overlapping and non-overlapping targets, so as to enhance the positioning precision of the network, which is principally suitable for the detection of sparsely distributed disease spots in the early stage of rice false smut.

### 3.9 Stability analysis and reproducibility with different random seeds

In this study, three independent random seeds were set for repeated validation experiments, and the evaluation metrics mean ± standard deviation of the three main benchmark models were compared. The results demonstrate that the number of parameters in the improved Rice-Smut model is 2.41 M, which is the lowest among all models. In addition, under the condition of 6.20 GFLOPs, the network attains a mean Average Precision of 0.7437 ± 0.0137 and a recall of 0.6780 ± 0.0035. Experimental data show that the enhancement of model performance is not due to random noise, but rather to the reproducibility and robustness of the proposed model. Under the premise of both efficiency and cost, the model solves the problem of real-time detection under complex field conditions, as detailed in **Table 6**.

**Table 6:** Model stability and reproducibility analysis (Mean ± SD).

Model	P	R	mAP@50	Params	FPS	GFLOPs
YOLOv8	78.47 ± 3.44	63.20 ± 2.84	72.87 ± 1.00	3.01 ± 0.00	158.40 ± 0.10	8.10 ± 0.00
YOLOv11	74.77 ± 3.15	66.03 ± 2.31	71.23 ± 0.38	2.58 ± 0.00	144.93 ± 0.25	6.30 ± 0.00
Rice-Smut(**ours**)	76.17 ± 2.74	67.80 ± 0.35	74.37 ± 1.37	2.41 ± 0.00	80.80 ± 1.41	6.20 ± 0.00

The bold values indicate the best-performing results for each evaluation metric.

### 3.10 Visual experiment

**Figure 12:**
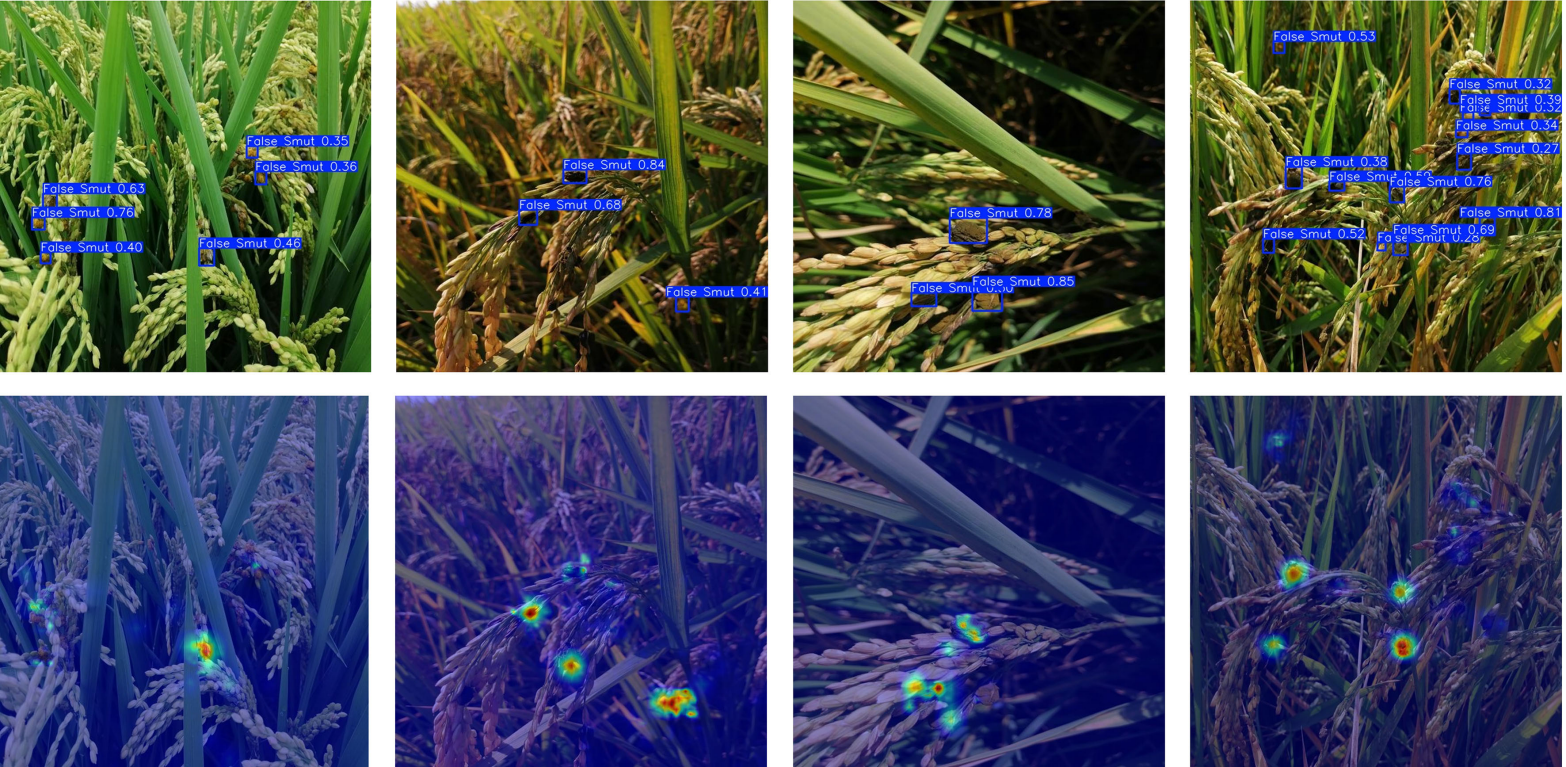
Corresponding visualized heatmap.

To clearly illustrate the ability of the Rice-Smut model in feature extraction and precise localization of lesions, this study uses the Grad-CAM algorithm to visually analyze the detection results under complex visual field conditions based on the heatmaps, as shown in **Figure 12**. Grad-CAM acts on the high-level feature maps of the backbone and neck sections of the model, and calculates the prediction confidence of the category as the goal, so as to generate the activation heatmap of the area related to the category.

Robustness analysis under complex environmental conditions: In the actual field environment, the disease lesion detection of rice false smut is easily disturbed by complex background noise, which leads to false positives. Despite the presence of significant interference factors such as overlap of leaf occlusion, light changes, and dense intertwined spikes with similar texture features in the preceding three columns of images, the activation value of the model in non-lesion areas, such as leaf and background areas, is still close to zero, which is shown in dark blue in the heatmap. The high response region of model feature extraction always focuses on the lesion region and presents a warm color region. This shows that the model has excellent disease feature selection ability and can precisely capture the color and texture characteristics of disease spots, thus showing good noise suppression and anti-interference capabilities. Recognition capability of dense small targets: During the incipient phase of infection, the size of disease spots is small and scattered within the dense spike structure. The observation results show that the highlighted active region of the model is highly consistent with the location of the lesion within the target bounding box. Even in the first and fourth columns of images containing very small lesion areas, the model can still effectively focus on the lesion features, which fully proves its powerful ability to capture dense small targets. Multi-scale perception: in the first, third, and fourth columns of the image, there are many lesions with different scales. Whether dealing with early small lesions or large mature lesions, the heatmap shows a high activation level, which effectively reduces the false negative rate of the network.

In conclusion, the visualization results indicate that the Rice-Smut architecture has stronger attention to the edge and overall representation of the lesion. The distribution of the heatmap shows a dot-like aggregation pattern, and this spatial selectivity shows that the model has effectively learned the deep semantic features for disease spot detection, so as to support its disease recognition ability under complex background conditions.

**Figure 13:**
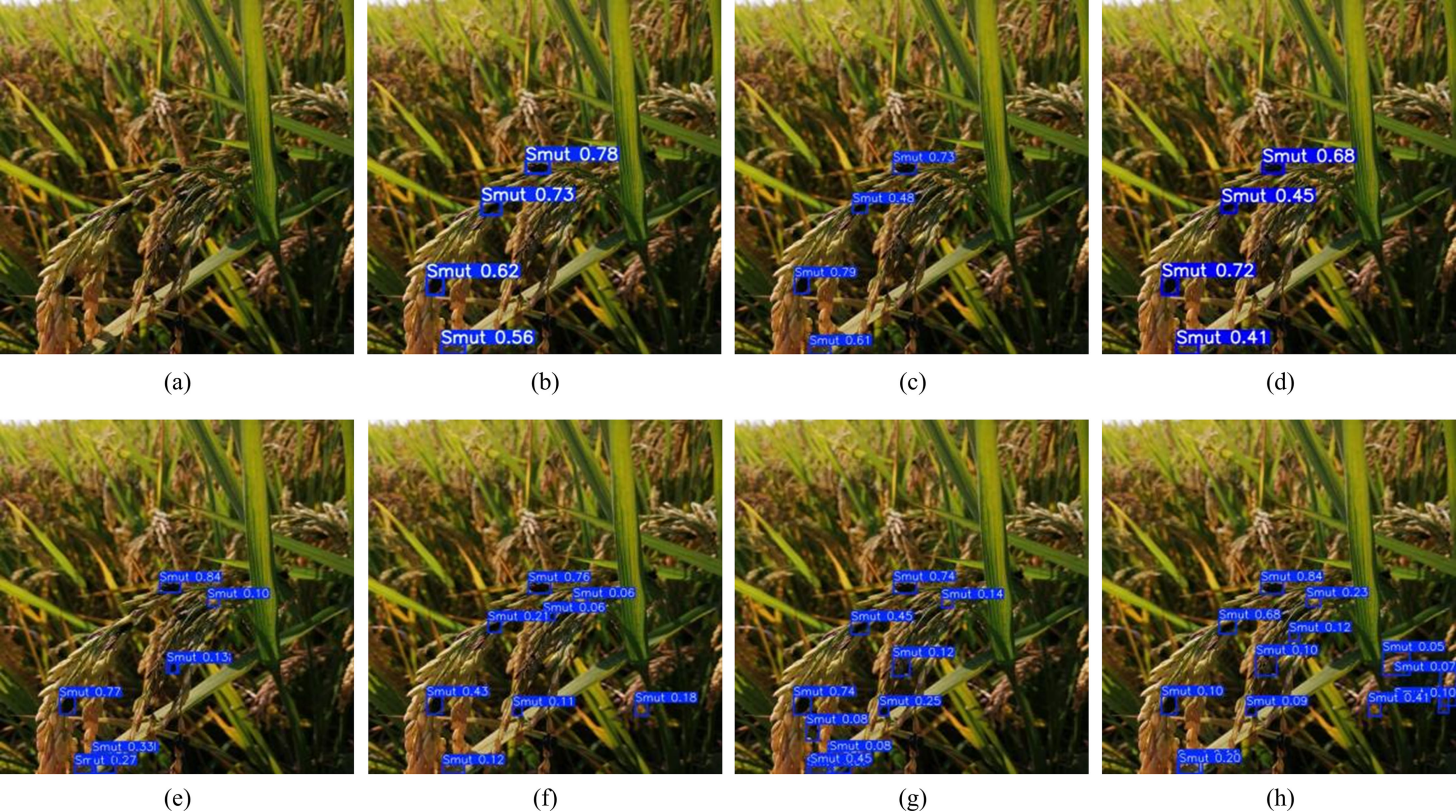
Comparative analysis of various detection models. **(A)** Original image; **(B)** YOLOv5 result; **(C)** YOLOv8 result; **(D)** YOLOv9-T result; **(E)** YOLOv10n result; **(F)** YOLOv11 result; **(G)** YOLOv12 result; **(H)** Rice-Smut result.

As demonstrated in **Figure 13**, the detection performance of Rice-Smut in complex natural scenes surpasses the YOLOv5–YOLOv12 foundational frameworks, especially in the number and confidence of rice false smut disease identification.

**Figure 14:**
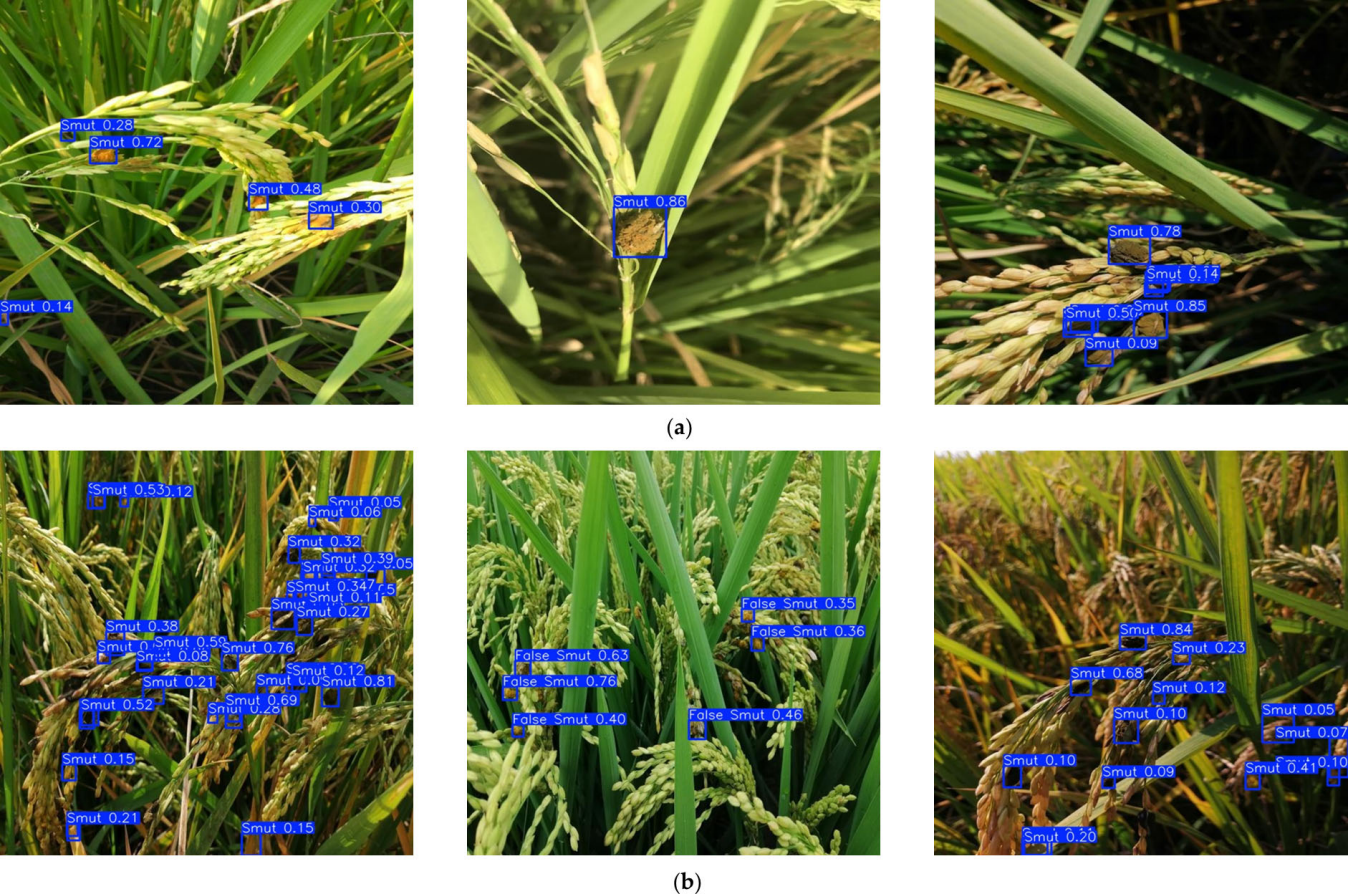
The Rice-Smut’s detection effect in real complex field conditions. **(A)** Under conditions of natural light changes; **(B)** In complex conditions of overlapping, dense small targets and occlusion of background interference target areas.

**Figure 14** shows the detection ability of the Rice-Smut model for rice false smut under complex field conditions. It provides shelter, changes light, and interferes with the background interference. (**Figure 14A**) shows the detection results under complex lighting conditions, and the confidence of the model in identifying the lesion is as high as 0.86. At the same time, (**Figure 14B**) shows the performance of the model in more complex scene conditions, such as dense rice panicle structure, overlapping disease spots, and background noise.

Despite these difficulties, the model can still sustain a good predictive precision, and its confidence score is mostly above 0.6. The experimental outcomes indicate that the refined model shows good generalization ability and detection performance in complex natural scenes, which makes it have a good application prospect in the actual farmland.

### 3.11 Analysis of detection failures

**Figure 15:**
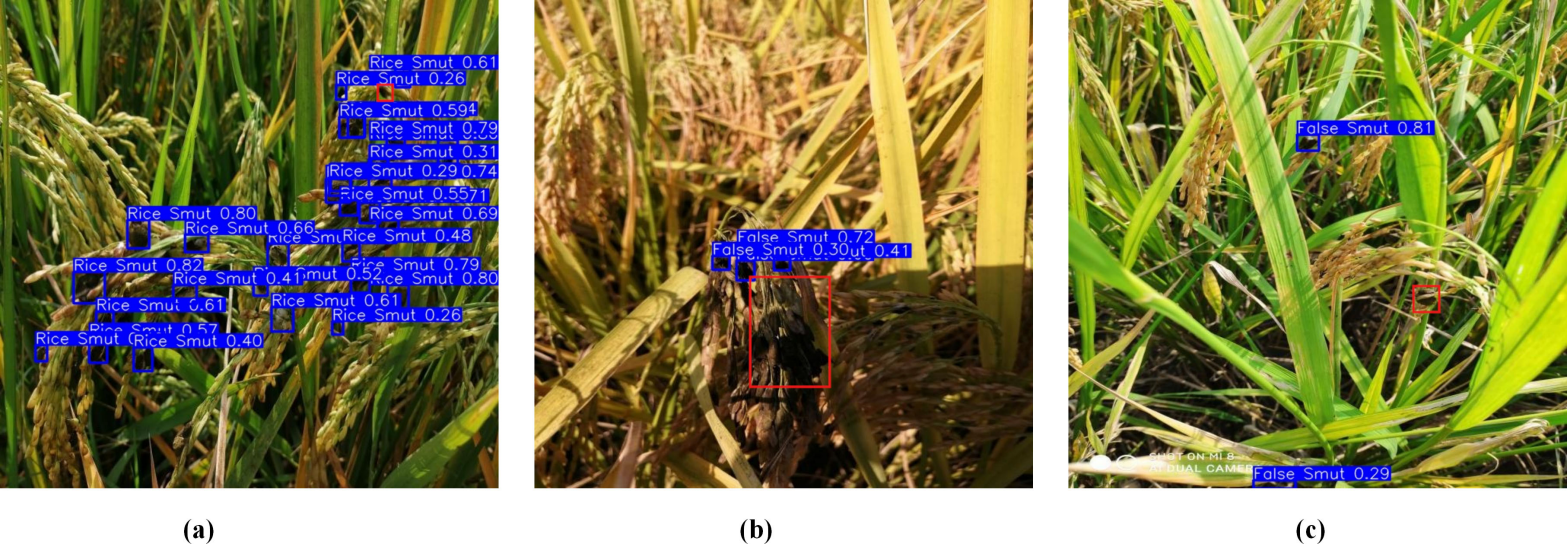
Analysis of detection failures. **(A)** Lesions appear as densely distributed small targets; **(B)** Lesions exhibit scenarios with drastic lighting variations; **(C)** Lesions present situations of occlusion.

Although the Rice-Smut model has excellent overall performance, it still has some limitations in complex field conditions. Under extreme conditions, such as confronting dense small targets, drastic illumination changes, and severe occlusion, the model may still exhibit missed detections and false positives. The model still has some shortcomings in capturing dense small target lesions, distinguishing multi-scale targets, and suppressing background interference. In addition, we strive to enhance the inference efficiency of the network, reduce the false positive rate, and strengthen the stability of the framework in the actual recognition scene. These limitations indicate that there is still enough potential for improvement in the network under extremely complex field environment. In the future, the detection capability of the model can be further modified by supplementing sample data and model optimization, as shown in **Figure 15**.

### 3.12 Design of real-time detection system for rice false smut on mobile terminal

**Figure 16:**
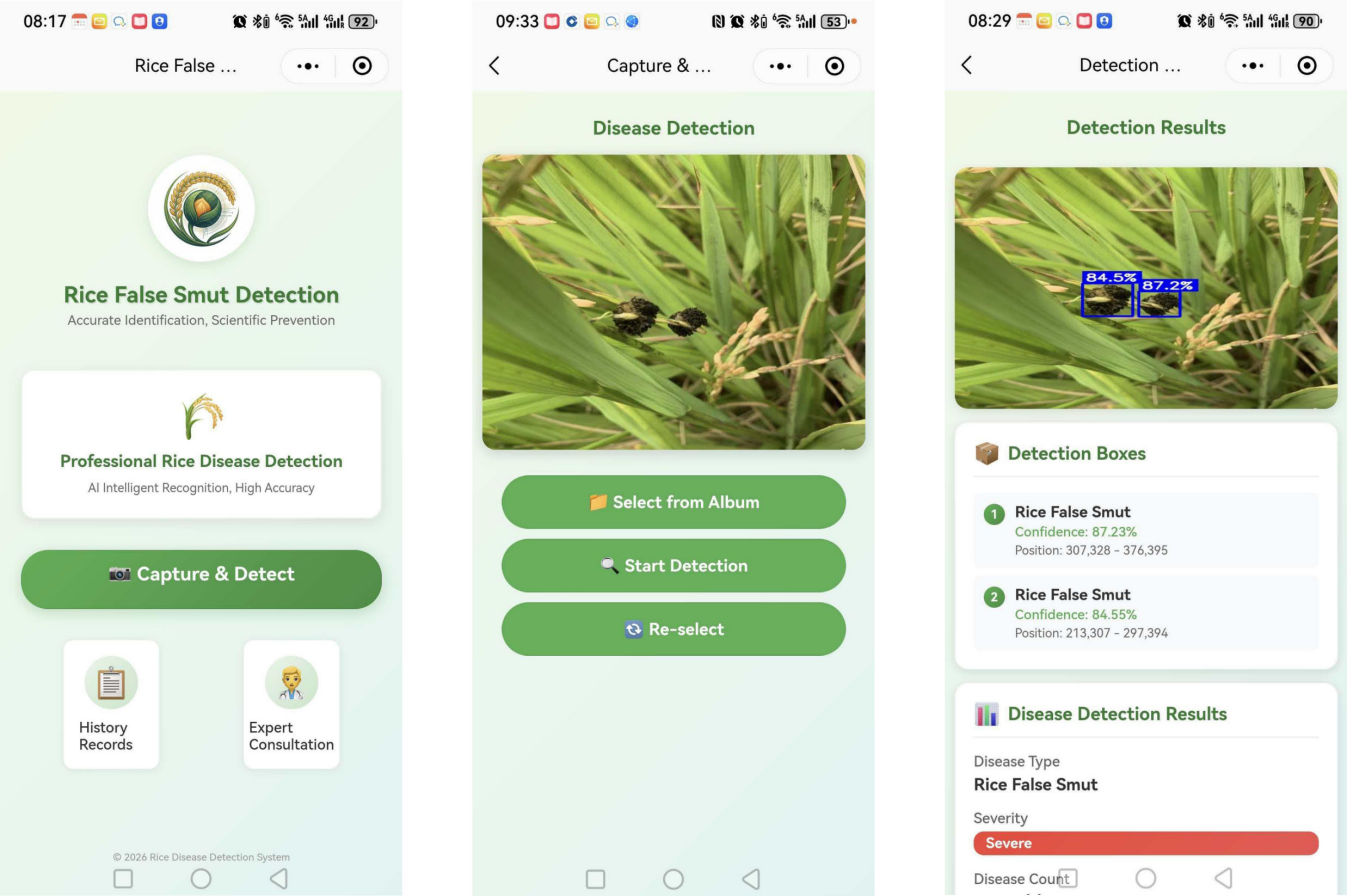
Main function interface of real-time detection of Rice False Smut based on mobile terminal.

This study built a mobile terminal application for real-time detection of rice false smut developed using Uniapp. The front-end uses Uniapp and Vue3.js, and the back-end uses FastAPI and the ONNX Runtime for cloud inference. The specific data processing workflow is as follows: first, the rice images collected in the field are manually labeled and utilized for training. The best weights derived from the training are exported to the ONNX format and deployed to a Tencent Cloud server to achieve efficient inference services. Then, agricultural users take photos on the spot or select photos from albums via the mobile app for real-time detection. The client sends a request through the API and parses the response data returned by the server. Finally, the image with the target detection boxes is dynamically rendered on the result interface, and the statistical information including the number of diseased panicles, maximum confidence, severity and control suggestions are summarized to facilitate field control decisions. The performance of the cloud-based inference system was evaluated using the Apifox API testing tool. Results indicated that the system achieved a total response time of 422.87 ms, with the actual model inference accounting for 150.87 ms, demonstrating robust computational efficiency. Under current experimental conditions, the system exhibits potential for near-real-time auxiliary detection of rice false smut in real-world agricultural settings. The main functions of the system are shown in **Figure 16**.

## 4 Discussion

From the perspective of agronomic application, rice false smut is usually covertly infected in the inner part of the rice panicle. Its early pathological characteristics are small and insignificant disease spots, and the detection process is susceptible to be interfered by leaf shading, the drastic change of light intensity and complex backgrounds, which increases the difficulty of visual detection. The improved diagnostic efficacy of the framework developed in this research under the conditions of dense small targets, multi-scale features and complex field noise is helpful to improve the early discrimination capability for rice false smut. This provides corresponding technical support for the meticulous management and mitigation of the pathology, helps to reduce the risk of misjudgment, and improves the stability of the detection results of rice false smut.

At the actual deployment level, this paper completed the preliminary engineering verification of the proposed model, and developed a mobile detection system for rice false smut based on Uniapp. The system supports field image acquisition through mobile devices, and realizes end-to-end real-time inference. It can quickly output the detection results of rice false smut disease, and provide auxiliary decision-making for farmers and agricultural technicians. These findings suggest that the model has good application potential in real field scenes, and further verifies the feasibility of deploying the model on hardware-limited platforms. However, at present, the verification of the mobile terminal detection system is still mainly based on single-region data and limited scene conditions, and has not been comprehensively evaluated considering the differences in geographic regions, planting modes and rice varieties, so its practical application generalization ability still needs to be further strengthened.

Although the model has achieved good detection results in experiments, its adaptability in complex field conditions still faces challenges. (1) Target recognition remains difficult, because background objects such as leaves, stems, soil, weeds, water and shadows may be highly similar to disease spots in color and shape, which can easily lead to cause false positives. (2) There are relatively few studies on rice panicle diseases, such as false smut. At present, both private and open-source rice false smut datasets lack high-quality data samples, and the number of samples available for training is limited. (3) The dataset used in this study is constrained. Because the early symptoms of the disease are faint white spots, which are often hidden inside the rice panicles, the number of training cases for the early stage is far less than those in the middle and late stages, and the uneven distribution of samples may pose a risk of overfitting and limit the reliability of the model. In addition, the applicability of the model across different geographical and climatic conditions and the diversity of rice varieties still faces certain challenges, which limits the generalization capability of the network in diverse field environments to a certain extent. (4) For actual field application scenarios, it is often necessary to balance missing detections and false positives. This method gives priority to ensuring the precision rate, aiming to reduce the false positive caused by the interference of complex conditions, thereby achieving effective early disease detection.

Finally, in order to address the above challenges, future work will focus on building large-scale and high-quality datasets covering different regions and in complex field environments from early to late growth stages, aiming to augment the resilience of model detection. In addition, it is planned to integrate the model into mobile applications or edge devices to enable the early identification and detection of rice false smut, reduce crop losses, and lay a solid technical foundation for smart agriculture.

## 5 Conclusion

This study presents a detection architecture for rice false smut in complex natural environments is proposed. The experimental findings demonstrate that the model achieved performance improvements in precision, recall and mAP@50 And other aspects have achieved performance improvement, and other evaluation indicators are stable. The effectiveness of the C3SC module, C2PSA-SE module and GIoU loss function was confirmed by comparative experiments, ablation analysis and visual verification, which enhanced the detection performance of the model as a whole. The architecture augments the feature representation ability of the backbone section by reducing redundant calculations in the spatial and channel dimensions. At the same time, the improved attention mechanism and bounding box regression strategy can effectively suppress noise interference, such as from illumination, occlusion and the background, and then improve the prediction precision of the network for lesion localization. In addition, by observing the heatmaps, it can be found that even under complex background conditions, the framework can still precisely focus on the texture area and irregular spherical structure of the lesion, showing good spatial selectivity.

Although the proposed method has achieved good performance under experimental conditions, the model has not been systematically verified in the large-scale field environments across different regions and varieties, thus, its generalization capability in diverse field environment settings still needs further validation. In addition, the current mobile terminal detection system is still in the preliminary application stage, and will need to be optimized in combination with specific application scenarios in the future to improve its stability in complex environments. In general, this work provides a lightweight technical solution with practical application potential for the intelligent detection of rice false smut. In the future, we will further enhance the generalization performance across large-scale regional datasets to facilitate the stable deployment of this method in actual agricultural production.

## Data Availability

The datasets generated for this study are available on request to the corresponding author.

## References

[B1] Ahad M. T. Li Y. Song B. Bhuiyan T. (2023). Comparison of CNN-based deep learning architectures for rice diseases classification. Artif. Intell. Agric. 9, 22–35. doi: 10.1016/j.aiia.2023.07.001. PMID: 38826717

[B2] Baite M. S. Khokhar M. K. Meena R. P. (2021). Management of false smut disease of rice: A review. IntechOpen. doi: 10.5772/intechopen.97329

[B3] Dey B. Haque M. M. U. Khatun R. Ahmed R. (2022). Comparative performance of four CNN-based deep learning variants in detecting Hispa pest, two fungal diseases, and NPK deficiency symptoms of rice (Oryza sativa). Comput. Electron. Agric. 202, 107340. doi: 10.1016/j.compag.2022.107340. PMID: 38826717

[B4] Fan Q. Chen R. H. Li B. (2025). Rice disease detection method based on multi-scale dynamic feature fusion. Front. Plant Sci. 16, 1543986. doi: 10.3389/fpls.2025.1543986. PMID: 40433155 PMC12106424

[B5] He L. Zhou Y. Liu L. Ma J. (2024). Research and application of YOLOv11-based object segmentation in intelligent recognition at construction sites. Buildings 14, 3777. doi: 10.3390/buildings14123777. PMID: 30654563

[B6] Hu J. Shen L. Sun G. (2018). “ Squeeze-and-excitation networks”, in: Proceedings of the 2018 IEEE/CVF Conference on Computer Vision and Pattern Recognition, 7132–7141.

[B7] Jiang Z. Dong Z. Jiang W. Yang Y. (2021). Recognition of rice leaf diseases and wheat leaf diseases based on multi-task deep transfer learning. Comput. Electron. Agric. 186, 106184. doi: 10.1016/j.compag.2021.106184. PMID: 38826717

[B8] Jiang F. Lu Y. Chen Y. Cai D. Li G. (2020). Image recognition of four rice leaf diseases based on deep learning and support vector machine. Comput. Electron. Agric. 179, 105824. doi: 10.1016/j.compag.2020.105824. PMID: 38826717

[B9] Khanal S. Gaire S. P. Zhou X. G. (2023). Kernel smut and false smut: The old-emerging diseases of rice—A review. Phytopathology 113, 931–944. doi: 10.1094/PHYTO-06-22-0226-RVW. PMID: 36441871

[B10] Li J. Wen Y. He L. (2023). “ Scconv: Spatial and channel reconstruction convolution for feature redundancy”, in: Proceedings of the IEEE/CVF Conference on Computer Vision and Pattern Recognition, 6153–6162.

[B11] Li H. Zhao F. Xue F. Wang J. Liu Y. Chen Y. . (2025). Succulent-YOLO: Smart UAV-assisted succulent farmland monitoring with CLIP-based YOLOv10 and Mamba computer vision. Remote Sens. 17, 2219. doi: 10.3390/rs17132219. PMID: 30654563

[B12] Liu G. Di J. Wang Q. Zhao Y. Yang Y. (2025). An enhanced and lightweight YOLOv8-based model for accurate rice pest detection. IEEE Access 13, 91046–91064. doi: 10.1109/ACCESS.2025.3569819. PMID: 25079929

[B13] Liu J. Wang X. (2021). Plant diseases and pests detection based on deep learning: a review. Plant Methods 17, 22. doi: 10.1186/s13007-021-00722-9. PMID: 33627131 PMC7903739

[B14] Liu Z. Wang J. Wu H. Xue F. Qin Z. Sun S. . (2026). Water-aware real-time detection of floating plastic debris via an enhanced YOLOv13 framework for aquatic pollution monitoring. Expert Syst. Appl. 313, 131552. doi: 10.1016/j.eswa.2026.131552. PMID: 38826717

[B15] Mukherjee R. Ghosh A. Chakraborty C. De J. N. Mishra D. P. (2025). Rice leaf disease identification and classification using machine learning techniques: A comprehensive review. Eng. Appl. Artif. Intell. 139, 109639. doi: 10.1016/j.engappai.2024.109639. PMID: 38826717

[B16] Pan P. Guo W. Zheng X. Hu L. Zhou G. Zhang J. (2023). Xoo-YOLO: a detection method for wild rice bacterial blight in the field from the perspective of unmanned aerial vehicles. Front. Plant Sci. 14, 1256545. doi: 10.3389/fpls.2023.1256545. PMID: 37936939 PMC10626997

[B17] Pan C. Wang S. Wang Y. Liu C. (2025). SSD-YOLO: a lightweight network for rice leaf disease detection. Front. Plant Sci. 16, 1643096. doi: 10.3389/fpls.2025.1643096. PMID: 40901551 PMC12399686

[B18] Rezatofighi H. Tsoi N. Gwak J. Sadeghian A. Reid I. Savarese S. (2019). “ Generalized intersection over union: A metric and a loss for bounding box regression”, in: Proceedings of the 2019 IEEE/CVF Conference on Computer Vision and Pattern Recognition (CVPR), 658–666.

[B19] Tian Y. Ye Q. Doermann D. S. (2025). YOLOv12: Attention-centric real-time object detectors. arXiv. Available online at: https://arxiv.org/abs/2502.12524. Preprint.

[B20] You S. Li B. Chen Y. Ren Z. Liu Y. Wu Q. . (2025). Rose-Mamba-YOLO: an enhanced framework for efficient and accurate greenhouse rose monitoring. Front. Plant Sci. 16, 1607582. doi: 10.3389/fpls.2025.1607582. PMID: 40655551 PMC12245864

[B21] Yusuf H. M. Yusuf S. A. Abubakar A. H. Abdullahi M. Hassan I. H. (2024). A systematic review of deep learning techniques for rice disease recognition: Current trends and future directions. Franklin Open 8, 100154. doi: 10.1016/j.fraope.2024.100154. PMID: 38826717

[B22] Zhao F. Ren Z. Wang J. Wu Q. Xi D. Shao X. . (2025a). Smart UAV-assisted rose growth monitoring with improved YOLOv10 and Mamba restoration techniques. Smart Agric. Technol. 10, 100730. doi: 10.1016/j.atech.2024.100730. PMID: 38826717

[B23] Zhao F. Xu D. Ren Z. Shao X. Wu Q. Liu Y. . (2025b). Mamba-based super-resolution and semi-supervised YOLOv10 for freshwater mussel detection using acoustic video camera: A case study at Lake Izunuma, Japan. Ecol. Inform., 103324. doi: 10.1016/j.ecoinf.2025.103324. PMID: 38826717

[B24] Zhou Y. Fu C. Zhai Y. Li J. Jin Z. Xu Y. (2023). Identification of rice leaf disease using improved ShuffleNet V2. Comput. Mater. Contin. 75 (2), 4501–4517. doi: 10.32604/cmc.2023.038446

